# Supplementary material for: Effectiveness and cost-effectiveness of basic versus biofeedback-mediated intensive pelvic floor muscle training for female stress or mixed urinary incontinence: protocol for the OPAL randomised trial
Source: BMJ Open. 2019 Feb 8;9(2):e024153. doi: 10.1136/bmjopen-2018-024153 (PMC6411252; doi:10.1136/bmjopen-2018-024153)
Supplement: Author's manuscript [file bmjopen-2018-024153.draft_revisions.pdf]

BMJ Open

BMJ Open is committed to open peer review. As part of this commitment we make the peer review history of every article we publish publicly available.

When an article is published we post the peer reviewers' comments and the authors' responses online. We also post the versions of the paper that were used during peer review. These are the versions that the peer review comments apply to.

The versions of the paper that follow are the versions that were submitted during the peer review process. They are not the versions of record or the final published versions. They should not be cited or distributed as the published version of this manuscript.

BMJ Open is an open access journal and the full, final, typeset and author-corrected version of record of the manuscript is available on our site with no access controls, subscription charges or pay-per-view fees (http://bmjopen.bmj.com).

If you have any questions on BMJ Open's open peer review process please email editorial.bmjopen@bmj.com

BMJ Open

Effectiveness and cost-effectiveness of basic versus biofeedback-mediated intensive pelvic floor muscle training for female stress or mixed urinary incontinence: protocol for the OPAL (Optimising Pelvic floor Exercises to Achieve Long-term benefits) randomised trial

Journal:	*BMJ Open*
Manuscript ID	bmjopen-2018-024153
Article Type:	Protocol
Date Submitted by the Author:	23-May-2018

Complete List of Authors:	Hagen, Suzanne; Glasgow Caledonian University<br>McClurg, Doreen; Glasgow Caledonian University, NMAHP RU<br>Bugge, Carol; University of Stirling, School of Health Sciences and Sport<br>Hay-smith, Jean; University of Otago Wellington, Rehabilitation Teaching and Research Unit<br>Dean, Sarah; PenCLAHRC University of Exeter Medical School,<br>Elders, A; Glasgow Caledonian University School of Health and Life Sciences, NMAHP Research Unit ; Glasgow Caledonian University<br>Glazener, Cathryn; University of Aberdeen, Health Services Research Unit<br>Abdel-fattah, Mohamed; University of Aberdeen, Obstetrics and Gynaecology<br>Agur, Wael; University of Glasgow, School of Medicine<br>Booth, Jo; Glasgow Caledonian University<br>Guerrero, Karen; NHS Greater Glasgow and Clyde<br>Norrie, John; Edinburgh Clinical Trials Unit, University of Edinburgh No. 9, Bioquarter<br>Kilonzo, M; University of Aberdeen<br>McPherson, Gladys; University of Aberdeen Institute of Applied Health Sciences<br>McDonald, Alison; University of Aberdeen, Health Services Research Unit<br>Stratton, Susan; Glasgow Caledonian University<br>Sergenson, Nicole; Glasgow Caledonian University<br>Grant, Aileen; Robert Gordon University, School of Nursing and Midwifery<br>Wilson, Lindsey; Patient advocate

Keywords:	urinary incontinence, pelvic floor muscle training, biofeedback, electromyography, adherence, long-term

SCHOLARONE™
Manuscripts

Title page

Effectiveness and cost-effectiveness of basic versus biofeedback-mediated intensive pelvic floor muscle training for female stress or mixed urinary incontinence: protocol for the OPAL (Optimising Pelvic floor Exercises to Achieve Long-term benefits) randomised trial

- Corresponding author.

Professor Suzanne Hagen
Nursing, Midwifery and Allied Health Professions Research Unit Glasgow
Caledonian University
Cowcaddens Road, Glasgow G4 0BA
0141 331 8104
s.hagen@gcu.ac.uk

- Co-authors.

Doreen McClurg
Nursing, Midwifery and Allied Health Professions Research Unit,
Glasgow Caledonian University,
Glasgow, UK

Carol Bugge
Faculty of Health Sciences and Sport,
University of Stirling,
Stirling, UK

Jean Hay-Smith
Department of Medicine,
University of Otago,
Dunedin, New Zealand

Sarah Dean
University of Exeter Medical School,
Exeter, UK

Andrew Elders
Nursing, Midwifery and Allied Health Professions Research Unit,
Glasgow Caledonian University,
Glasgow, UK

Cathryn Glazener
Health Services Research Unit,
University of Aberdeen,
Aberdeen, UK

Mohamed Abdel-fattah
Division of Applied Health Sciences,
University of Aberdeen,
Aberdeen, UK

For peer review only

For peer review only - http://bmjopen.bmj.com/site/about/guidelines.xhtml

Wael Agur
NHS Ayrshire and Arran University Hospital,
Crosshouse Hospital,
Kilmarnock, UK

Jo Booth
School of Health and Life Sciences,
Glasgow Caledonian University,
Glasgow, UK

Karen Guerrero
NHS Greater Glasgow and Clyde,
Queen Elizabeth Hospital,
Glasgow, UK

John Norrie
Edinburgh Clinical Trials Unit,
University of Edinburgh,
Edinburgh, UK

Mary Kilonzo
Health Economics Research Unit,
University of Aberdeen,
Aberdeen, UK

Gladys McPherson
The Centre for Healthcare Randomised Trials,
Health Services Research Unit,
University of Aberdeen,
Aberdeen, UK

Alison McDonald
The Centre for Healthcare Randomised Trials,
Health Services Research Unit,
University of Aberdeen,
Aberdeen, UK

Susan Stratton
Nursing, Midwifery and Allied Health Professions Research Unit,
Glasgow Caledonian University,
Glasgow, UK

Nicole Sergenson
Nursing, Midwifery and Allied Health Professions Research Unit,
Glasgow Caledonian University,
Glasgow, UK

Aileen Grant

For peer review only

For peer review only - http://bmjopen.bmj.com/site/about/guidelines.xhtml

School of Nursing and Midwifery,
Robert Gordon University,
Aberdeen. UK

Lyndsay Wilson,
Kilwinning, UK

WORD COUNT: 3327

KEYWORDS

Urinary incontinence; pelvic floor muscle training; biofeedback; electromyography; adherence; long-term

For peer review only

For peer review only - http://bmjopen.bmj.com/site/about/guidelines.xhtml

Effectiveness and cost-effectiveness of basic versus biofeedback-mediated intensive pelvic floor muscle training for female stress or mixed urinary incontinence: protocol for the OPAL randomised trial

Suzanne Hagen, Doreen McClurg, Carol Bugge, Jean Hay-Smith, Sarah Dean, Andrew Elders, Cathryn Glazener, Mohamed Abdel-fattah, Wael Agur, Jo Booth, Karen Guerrero, John Norrie, Mary Kilonzo, Gladys McPherson, Alison McDonald, Susan Stratton, Nicole Sergenson, Aileen Grant, Lyndsay Wilson

ABSTRACT

Introduction: Accidental urine leakage is a distressing problem that affects around one in three women. The main types of urinary incontinence (urinary incontinence) are stress, urgency and mixed, with stress being most common. Current UK guidelines recommend that women with UI are offered at least three months of pelvic floor muscle training (PFMT). There is evidence that PFMT is effective in treating UI, however it is not clear how intensively women have to exercise to give the maximum sustained improvement in symptoms, and how we enable women to achieve this. Biofeedback is an adjunct to PFMT that may help women exercise more intensively for longer, and thus may improve continence outcomes when compared to PFMT alone. A Cochrane review was inconclusive about the benefit of biofeedback, indicating the need for further evidence.

Methods and analysis: This multicentre randomised controlled trial will compare the effectiveness and cost-effectiveness of PFMT versus biofeedback-mediated PFMT for women with stress UI or mixed UI. The primary outcome is UI severity at 24 months after randomisation. The primary economic outcome measure is incremental cost per quality-adjusted life-year at 24 months. Six hundred women will be randomised. An intention-to-treat analysis of the primary outcome will estimate the mean difference between the trial groups at 24 months using a general linear mixed model adjusting for minimisation covariates and other important prognostic covariates, including the baseline score.

Ethics and dissemination: Approval granted by the West of Scotland Research Ethics Committee 4 (16/LO/0990). Written informed consent will be obtained from participants. Serious adverse events will be reported to the data monitoring and ethics committee, the ethics committee and trial centres as required. A SPIRIT checklist and figure are available for this protocol. The results will be published in international journals and included in the relevant Cochrane Review.

Registration: ISRCTN 57746448.

Keywords: urinary incontinence; pelvic floor muscle training; biofeedback; electromyography; adherence; long-term

STRENGTHS AND LIMITATIONS

1. The main strengths of our trial are its size, rigour, and pragmatic design.
2. The intervention being evaluated is relevant to UK NHS practice, and potentially to other similar health systems worldwide. It was developed to include explicit, named, behaviour change strategies to support adoption and maintenance of PFMT, and could be tailored to individual women's circumstances.
3. The outcome measures used within the trial are woman-centred which will ensure that the findings are relevant to those with the condition. We ask women about the severity of their urine leakage symptoms using a validated questionnaire and also about the impact the symptoms have on their quality of life.
4. The research includes an extensive longitudinal qualitative case study and a process evaluation that will provide rich data from both women and healthcare professionals about their experiences of the intervention and being involved in the trial, providing insight into how exercise therapy may be intensified to optimise treatment adherence and longer term outcomes. These components are described in a companion paper.

1. The main limitation of the trial is that the effect of the pelvic floor muscle training intervention delivered in both

For peer review only - http://bmjopen.bmj.com/site/about/guidelines.xhtml

groups (comprising a robust, evidence-based protocol with six supervised appointments) is likely to be strong. Thus the impact of adding biofeedback will need to be sizeable in order for a difference between groups to be detected.

2. Another potential limitation is that biofeedback equipment is advancing rapidly and biofeedback units are now available which incorporate bluetooth and smart phone technology. These new features, not available in this trial, may mean that women are less constrained in using such devices, leading to an enhanced effect.
3. There is the possibility that women randomised to the pelvic floor muscle training group could also access biofeedback, as devices are available to buy, and this could potentially dilute the difference between groups.

INTRODUCTION

Urinary incontinence (UI), defined as any involuntary loss of urine,[1] is a common condition in women. The prevalence of UI depends on the definition: using a broad definition, a range of between 5% and 69% is reported, with most studies in the range 25% to 45%.[1] The main types of UI are stress, urgency and mixed, with stress being most prevalent (accounts for around half of all UI), followed by mixed UI (stress and urgency combined) (most studies report 7.5-25%), and fewer having urgency UI alone (most studies report 1-7%). The cost to the UK NHS annually of treating clinically significant UI in women was estimated in the Leicestershire MRC Incontinence Study[2] as £233M, not including the personal costs borne by the women which were estimated to be £178M in the same study.[3] UI significantly impacts on daily living for the majority of women, with an associated increased prevalence of major depression.[4] Among older women, social isolation, psychological distress[5] and increased risk of admission to long-term care institutions[6] feature highly. Thus UI is prevalent and costly to women, the NHS and society both financially and in terms of physical and mental wellbeing.

Currently, supervised pelvic floor muscle training (PFMT) of at least three months' duration is the first-line treatment for stress and mixed UI.[7] PFMT involves the regular practice of repeated voluntary pelvic floor muscle contractions, with sufficient exercise progression, in order to produce a training effect on the muscles. The aim of a PFMT programme is to increase the strength of the muscles, to build up muscle volume and thus improve structural support; to increase contraction endurance; to improve muscle resting tone; to improve muscle recruitment through improved nerve function and properties of muscle fibres; and improve cognitive awareness of body posture and a relaxed versus an un-relaxed state of the pelvic floor. In addition, a PFMT program involves instruction on contracting and relaxing the pelvic floor muscles to improve coordination and functional use (termed the 'Knack') to occlude the urethra during physical activities that increase abdominal pressure and precipitate UI. Pelvic floor muscle contraction can also be used to inhibit a bladder contraction at the time of urgency as a method of urge suppression.

To produce improvements in muscle strength and endurance the basic physiological principles must be adhered to:[8] overload (muscles need to perform more work than usual to the point of fatigue); specificity (muscles must be trained with exercise or physical activity that replicates as closely as possible the functional movement required); maintenance and reversibility (benefits of the exercise will reverse if they are not undertaken on a regular basis). There is evidence to suggest that for effective resistance training in skeletal muscles in adults, two to four sets of 8 to 12 slow to moderate velocity, moderate to maximal intensity contractions be performed per day, 2 to 3 days per week with progression over 16 weeks.[9,10]

Biofeedback is commonly used as an adjunct to PFMT. Biofeedback is the technique by which information about a normally unconscious physiological process is presented to the patient and/or the therapist as a visual, auditory or tactile signal.[11] Electromyography (EMG) is the study of minute electrical potentials produced by depolarisation of muscle membrane.[12] In EMG biofeedback, electrical activity arising from muscle activity (during exercise and voluntary effort) is recorded in microvolts and displayed as a visual or auditory signal for both patient and therapist to view. Within a PFMT programme, an internal vaginal probe is used to record electrical information from pelvic

For peer review only - http://bmjopen.bmj.com/site/about/guidelines.xhtml

floor muscles through surface recordings. The probe is connected by cables to a biofeedback unit. Handheld units with a small visual display screen are available for home use. The display provides a visual representation of the muscles contracting and relaxing, allowing monitoring of strength, endurance and repetitions. The effectiveness of using biofeedback to augment PFMT for UI in women has been the subject of a Cochrane review, the conclusions of which were that women who had PFMT including biofeedback were more likely to report cure or improvement of UI than those who had PFMT without biofeedback, but this finding was potentially confounded by more health professional contact and supervised treatment time in the biofeedback groups.[13] Biofeedback in general is considered an evidence-based Behaviour Change Technique (BCT) according to the BCT Taxonomy.[14]

The effect of PFMT relies firstly on sufficient exercise being undertaken for a long enough period to strengthen and hypertrophy the muscles, and secondly the continuation of sufficient exercise so that strength is maintained.[15] Thus to intensify PFMT, adherence must be maximised both initially (i.e. uptake or adoption of the exercise programme) and then continued over the longer-term (maintenance). Based on current evidence, it is not clear if the apparent benefit of biofeedback can be attributed to the biofeedback or to some other variable such as more health professional contact in those women receiving biofeedback.[13,16] A common problem in previous trials was the failure to clearly state the purpose of biofeedback, or to describe the intervention protocol.[13] Thus, it was not clear if the way the biofeedback was used could theoretically or in practice qualitatively change the intensity or effectiveness of the PFMT. In our trial the purpose of biofeedback is twofold: to enhance uptake initially as women will know they are performing the correct technique, and to support ongoing exercise training adherence over the longer-term.

The aim of this trial is to determine the effectiveness and cost-effectiveness of PFMT compared to biofeedback-mediated PFMT for the treatment of stress or mixed UI. Both trial groups will have the same PFMT programme and same amount of health professional contact to establish if the addition of biofeedback improves incontinence outcomes.

METHODS AND ANALYSIS

This trial design is a parallel group multicentre randomised controlled trial, with nested longitudinal qualitative case study and process evaluation (Figure 1), set in UK community, outpatient and primary care settings, where continence care is usually provided, comparing effectiveness and cost-effectiveness of PFMT versus biofeedback-mediated PFMT for women with stress or mixed UI. The trial is assessing superiority of PFMT plus biofeedback over PFMT alone. The start date is 1 September 2013 and finish date 30 November 2018.

Trial groups

Participants in this trial will be randomly assigned to either PFMT or biofeedback-mediated PFMT. Allocation will be generated by a web-based programme developed and implemented by The Centre for Healthcare Randomised Trials, situated remotely from the trial office and recruiting centres.

Prior to randomisation, as part of the screening process, a visual and digital assessment of the woman's vagina and pelvic floor muscles will be carried out to provide accurate knowledge of the condition of the perineum and vagina (including areas of pain or increased tone), and the woman's capacity for contracting and relaxing her pelvic floor muscles.

Control group (PFMT)

After randomisation, women will be taught by a trained therapist (nurse or physiotherapist) the correct exercise technique and this will be confirmed on digital palpation with the woman in the supine position. Women will be encouraged to become aware of the sensation or feeling of contracting and holding the appropriate muscles, and also of relaxing them.

For peer review only - http://bmjopen.bmj.com/site/about/guidelines.xhtml

Women in the PFMT group will be offered six appointments with the trained therapist over a 16-week period at weeks 0, 1, 3, 6, 10 and 15. During these appointments the PFMT programme will be delivered and home exercise prescribed.

The initial PFMT programme will be identified and agreed between the individual woman and her therapist over the first and second appointment, according to the woman's ability. The exercise programme will be practised during the appointments to allow the therapist to assess progress and adjust the programme as necessary. Home exercise will be prescribed after the first appointment, or as soon as the therapist confirms a correct technique has been achieved.

There are two main components to the PFMT protocol: resistance training and counterbracing (also called 'The Knack'[17]). Initial exercise prescription in terms of number and type of contractions, length of hold, number of sets, position, will be informed by the PERFECT assessment.[18] The programme is progressed (e.g. increasing the number of repetitions by one, or duration of hold by one second, each week) according to the woman's ability to reach her individualised goal (e.g. 3 exercise days per week with 3 exercise sets per day and a minimum of 3 minutes between sets, a set comprising 10 repetitions held for 10 seconds each with 10 seconds rest between, followed by 10 fast contractions). BCTs are built in to the PFMT protocol: a set of core CBTs are to be used if possible at each appointment (e.g. agree and record PFMT action plan in new exercise diary) and a further set of optional CBTs are to be used at the therapist's discretion (e.g. discuss pros and cons of doing PFMT). The use of the CBTs is recorded by therapists in a checklist.

No biofeedback equipment is used in the control group, although verbal feedback based on digital vaginal palpation is permitted.

Experimental group (PFMT plus biofeedback)

Biofeedback is included in the experimental group in addition to all that is described above for the control group (both PFMT and BCT protocols).

Participants randomised to receive PFMT plus biofeedback will have a biofeedback protocol incorporated into their PFMT appointments, and will be given a biofeedback unit to use during home exercise sessions between appointments. The content of the biofeedback protocol is underpinned by the Information-Motivation-Behavioral Skills (IMB) model of behaviour change,[19] incorporating Social Cognitive Theory,[20] an evidence-based theory relating to self-efficacy and perseverance. It is hypothesised that self-efficacy for PFMT is enhanced because biofeedback offers information to women about a normally 'hidden' muscle activity. Biofeedback also supports motivation through providing audio and visual feedback (graphs on screen which can be printed off) on the 'effort/performance' of the muscle contraction, which facilitates tracking changes in muscle strength and performance, and enhances behavioural skills through improving the performance of a muscle contraction and the timing of a contraction to reduce leakage with increases in intra-abdominal pressure (e.g. during cough, sneeze, lift). In this way the biofeedback 'intensive' trial group incorporates behaviour change techniques additional to those in the control group, that aim to enhance self-efficacy.

Training in intervention delivery

All therapists delivering the trial interventions will receive training from the trial team on using the biofeedback units and associated software, implementing the PFMT, BCT and biofeedback protocols and completing the trial paperwork to ensure standardisation across centres. An Intervention Training Manual will be provided to each centre covering details of the intervention development and delivery for both the control and experimental groups. Practical sessions using the biofeedback units will be incorporated into the training and there will be a forum for

For peer review only - http://bmjopen.bmj.com/site/about/guidelines.xhtml

discussion with opportunities for questions. All centres will use the same model of biofeedback unit for clinic and home use.

Primary outcome measure: The severity of UI at 24 months is the primary outcome, as measured by the International Consultation on Incontinence Questionnaire - Urinary Incontinence Short Form (ICIQ-UI SF) which encompasses urinary leakage frequency, amount and interference with everyday life (total score 0-21, higher score indicates more severe UI).[21]

Secondary outcomes measures:

- Perceived improvement in UI (PGI-I)[22]
- Number with UI cured/improved (derived from the ICIQ-UI SF)[21]
- Uptake of surgery for UI
- Uptake of other treatment for UI
- Other urinary symptoms (ICIQ-FLUTS)[23]
- UI-specific quality of life (ICIQ-LUTSqol)[24]
- Prolapse symptoms (POP-SS)[25]
- Bowel symptoms (early version of ICIQ Bowel Short Form)
- Pelvic floor muscle function (Oxford scale,[26] ICS method[27])
- Self-efficacy for PFMT (PFME self-efficacy scale)[28]
- Adherence to PFMT (derived from exercise diary/follow-up questionnaire)
- Incremental cost per quality-adjusted life-year (EQ-5D-3L)[29]

Centre Selection

This trial will recruit from community, primary and secondary care settings across the UK. The first three centres will be identified and will start recruiting, providing an internal pilot. Experience of recruitment and data generated from these centres over three months will be used to fine-tune trial processes and protocols at new centres. Recruitment at other centres will be staggered with potentially more than 20 centres recruiting to the trial. Potential centres complete a feasibility questionnaire asking about: the number of women they could recruit and how they would be identified; available staff, facilities and time to commit to the trial; previous experience of undertaking trials; any challenges and risks anticipated. A selection will be made on this basis.

Participant Selection

Women attending for a first continence appointment, or for a first outpatient appointment where UI is the presenting complaint, will be identified in advance from clinic lists in each centre, over the trial recruitment period. An OPAL Participant Information Leaflet will be given to potentially eligible women either before or at their routine appointment. When a woman attends for her appointment with her continence specialist they will confirm a clinical diagnosis of stress UI or mixed UI (those with urgency UI alone will be excluded), and assess other trial eligibility criteria by undertaking a vaginal examination as part of their routine care.

Inclusion criteria

- Women 18 years of age or above, presenting with a new episode of stress or mixed UI, who are willing and suitable to be randomised.

Exclusion criteria

- Women who have urgency UI alone;

For peer review only - http://bmjopen.bmj.com/site/about/guidelines.xhtml

- Women who have had formal instruction in PFMT in the last year;
- Women who are unable to contract their pelvic floor muscles;
- Women who are pregnant or are less than 6 months postnatal;
- Women who have prolapse greater than stage II (>1cm below the hymen on Valsalva);
- Women who are currently having treatment for pelvic cancer;
- Women who have cognitive impairment affecting capacity to give informed consent;
- Women who have neurological disease (Multiple Sclerosis, Parkinson's Disease, Stroke, Motor Neurone Disease, Spinal Injury);
- Women with a known nickel allergy or sensitivity;
- Women who are currently participating in other research relating to their UI.

For peer review only

Randomisation

Women who are eligible and willing to take part will be randomised to either the experimental or control group via the automated computer randomisation application developed at the Centre for Healthcare Randomised Trials (CHaRT), University of Aberdeen. Allocation will be minimised on: type of UI (stress UI or mixed UI), centre, age (<50/≥50 years) and UI severity at baseline (ICIQ-UI SF score <13/≥ 13).[30]

Masking

Statistical analysis will be conducted by research staff with the group allocation masked, using study identity numbers only to identify women and questionnaires. Due to the nature of the interventions, group allocation cannot be concealed from the women, the therapists delivering the intervention or those entering data. The clinician undertaking 6-month pelvic floor muscle assessments will not be aware of the woman's group allocation.

Data collection and management

Data collection and entry is managed by experienced staff at the OPAL Trial Office situated in NMAHP Research Unit, Glasgow Caledonian University. Data are entered into the CHaRT web-based database. Trained therapists or research nurses at centres collect data on paper Case Report Forms at every participant appointment. Centre staff enter the screening and eligibility data directly onto the web-based database in order to randomise participants. Participants complete paper diaries throughout treatment and outcome questionnaires at baseline, 6, 12 and 24 months' post randomisation. Paper copies of Case Report Forms, diaries and questionnaires are returned by post to the OPAL Trial Office to be entered onto the database. Copies of Case Report Forms are also securely stored at each centre in accordance with local ethics protocols. Data from the longitudinal qualitative case study and process evaluation are gathered alongside and in addition to the main trial data and are described in the companion paper.

Statistical analysis and sample size

All analyses will be according to the Statistical Analysis Plan and Economic Analysis Plan that will be agreed in advance with the Project Management Group (PMG) and the Trial Steering Committee (TSC). A single main analysis will be performed at the end of the trial when 24-month follow up has been completed. An independent Data Monitoring and Ethics Committee (DMEC) will review confidential interim analyses of accumulating data at its discretion, at least annually. Analysis will be based on the intention-to-treat principle, such that women will be analysed in the groups to which they were allocated regardless of the intervention received.

The main effectiveness analysis will estimate the mean difference in the primary outcome (ICIQ-UI SF score) (and 95% confidence intervals) between the experimental and control groups at 24 months using a general linear mixed model, adjusting for minimisation covariates, therapist type and baseline score. Centre and therapist type will be fitted, where possible, as random effects. Missing outcome data will be estimated using multiple imputation

and included in a sensitivity analysis Secondary outcomes will be analysed in a similar manner using an appropriate generalised linear model. Complier average causal effect analysis of the primary outcome will be conducted to investigate the impact of non-compliance. Subgroup analyses by type of incontinence (stress UI or mixed UI), type of therapist (physiotherapist or nurse), participant age (<50/≥50 years) and baseline UI severity (ICIQ-UI SF score <13/≥ 13) are also planned.

The primary economic outcome measure of cost effectiveness is incremental cost per quality-adjusted life-year (QALY) at 24 months undertaken from the NHS perspective. QALYs will be based on responses to the EQ-5D-3L[30] and the ICIQ-LUTSqol.[24] Personal patient resource use data will be incorporated into the analysis to report on a wider perspective. Where appropriate the analysis of incremental costs, effectiveness and cost-effectiveness will be based on similar statistical models as those outlined in the statistical analysis section above. This 'within' trial analysis will include both deterministic and stochastic sensitivity analyses to explore statistical and other forms (e.g. around unit costs or the source of utility estimates) of uncertainty. Similar subgroup analysis will be performed in the economic analysis as defined in the statistical analysis if deemed relevant. If relevant an economic model will be developed to provide additional information for policy makers.

Assuming a clinically meaningful difference of 3 points on the ICIQ-UI SF score (e.g. change from leaking urine "once a day" to "never"), which is similar to the minimal clinically important difference of 2.5 reported in a study of older women,[31] and a conservative estimate for the SD of 10, a sample size of 234 per group would detect this difference (standardised effect size of 0.3) with 90% power at the 5% level of significance (2- sided alpha). Allowing for 22% drop out, we will randomise 300 women per group. With 600 subjects we will also have 90% power to detect important differences in secondary outcomes.

Safety measures

The OPAL trial involves treatments for UI which are well established in clinical practice, therefore adverse effects (although these are unlikely) will be those observed in everyday practice associated with the use of PFMT and biofeedback. In the OPAL trial all serious adverse events experienced by a research participant will be reported to the main research ethics committee if they occur within 30 days of the participant's last PFMT appointment and where in the opinion of the Chief Investigator and/or the Chair of the DMEC the event is related and unexpected.

Patient involvement

Women with experience of urinary incontinence are involved in the design, delivery and oversight of the trial through membership on the PMG and TSC. A lay summary of the findings will be sent to participants involved in the trial.

ETHICS AND DISSEMINATION

The trial will be conducted in accordance with the ICH GCP Note for Guidance on Good Clinical Practice. Favourable ethics opinion covering recruitment across all UK NHS centres was obtained from the West of Scotland REC 4 (approved 13th March 2013, reference number 13/WS/0048) and local R&D departments. Trial safety and progress will be overseen by the independent DMEC and the TSC. Meetings of these committees will take place annually.

Final trial results will be disseminated to the funding body, the NIHR Health Technology Assessment Programme. The trial results will be then submitted to peer-reviewed international academic journals and presented at international conferences. Results will also contribute to the relevant Cochrane review. Participants will be provided with a summary of the results.

REFERENCES

For peer review only - http://bmjopen.bmj.com/site/about/guidelines.xhtml

1.	Abrams P, Cardozo L, Wagg A, Wein A. Incontinence, 6th Edition. Health Publication Ltd 2017.
2.	Perry S, Shaw C, Assassa P, et al. An epidemiological study to establish the prevalence of urinary symptoms and felt need in the community: the Leicestershire MRC Incontinence Study. Leicestershire MRC Incontinence Study Team. *Journal of Public Health* 2000;22(3):427–34.
3.	Turner DA, Shaw C, McGrother CW, H.M. Dallosso, Cooper NJ. The MRC Incontinence Team. The cost of clinically significant urinary storage symptoms for community dwelling adults in the UK. *BJU Int* 2004;93(9):1246–52.
4.	Melville JL, Fan MY, Rau H, Nygaard IE, Katon WJ. Major depression and urinary incontinence in women: temporal associations in an epidemiologic sample. *Am J Obstet Gynecol* 2009;201:490el–490e7.
5.	Bognor HR, Gallo JJ, Sammel MD, Ford DE, Armenian HK, Eaton WW. Urinary incontinence and psychological distress in community-dwelling older adults. *J Am Geriatr Soc* 2002;50:489–495.
6.	Thom DH, Haan MN, Van Den Eeden SK. Medically recognised urinary incontinence and risks of hospitalisation, nursing home admission and mortality. *Age Ageing* 1997;26:367–374.
7.	Welsh A. Urinary incontinence–the management of urinary incontinence in women. National Collaborating Centre for Women's and Children's Health. The National Institute for Health and Clinical Excellence. National Institute for Health and Clinical Excellence, Royal College of Obstetricians and Gynaecologists. London: RCOG Press 2006.
8.	McArdle WD, Katch FI. Katch VL. Principles of Training. In Essentials of Exercise Physiology. Philadelphia: Lea and Febiger 1994:346–349.
9.	Garber CE, Blissmer B, Deschenes MR, Franklin BA, Lamonte MJ, Lee IM, Nieman DC, Swain DP. American College of Sports Medicine position stand. Quantity and quality of exercise for developing and maintaining cardiorespiratory, musculoskeletal, and neuromotor fitness in apparently healthy adults: guidance for prescribing exercise. *Med Sci Sports Exerc* 2011;43(7):1334–59.
10.	Kraemer WJ, Adams K, Cafarelli E, Dudley GA, Dooly C, Feigenbaum MS, Fleck SJ, Franklin B, Fry AC, Hoffman JR, Newton RU. American College of Sports Medicine position stand. Progression models in resistance training for healthy adults. *Med Sci Sports Exerc* 2002;34(2):364–80.
11.	Abrams P, Cardozo L, Fall M, Griffiths D, Rosier P, Ulmsten U, van Kerrebroeck P, Victor A, Wein A. The standardisation of terminology of lower urinary tract function: Report from the standardisation sub-committee of the International Continence Society. *Neurourol Urodyn* 2002;21(2):167–78.
12.	Siroky MB. Electromyograph of the perineal floor. *Urol Clin North Am* 1996;23(2):299–307.
13.	Herderschee R, Hay-Smith EJ, Herbison GP, Roovers JP, Heineman MJ. Feedback or biofeedback to augment pelvic floor muscle training for urinary incontinence in women. *Cochrane Libr* 2011 Jan 1.
14.	Michie S, Richardson M, Johnston M, Abraham C, Francis J, Hardeman W, Eccles MP, Cane J, Wood CE. The behavior change technique taxonomy (v1) of 93 hierarchically clustered techniques: building an international consensus for the reporting of behavior change interventions. *Ann Behav Med* 2013;46(1):81–95.
15.	Bø K. Pelvic floor muscle training is effective in treatment of female stress urinary incontinence, but how does it work? *Int Urogynecol J* 2004;15(2):76–84.
16.	Imamura M, Abrams P, Bain C, Buckley B, Cardozo L, Cody J, Cook J, Eustice S, Glazener CM, Grant A, Hay-Smith J. Systematic review and economic modelling of the effectiveness and cost-effectiveness of non-surgical treatments for women with stress urinary incontinence. *Health Technol Assess* 2010.
17.	Miller JM, Ashton-Miller JA, DeLancey JO. A pelvic muscle precontraction can reduce cough-related urine loss in selected women with mild SUI. *J Am Geriatr Soc* 1998;46(7):870–4.
18.	Laycock J, Jerwood D. Pelvic floor muscle assessment: the PERFECT scheme. *Physiotherapy*

For peer review only - http://bmjopen.bmj.com/site/about/guidelines.xhtml

	2001;87(12):631–42.
19.	Fisher JD, Fisher WA. The information-motivation-behavioral skills model. In: DiClemente R, Crosby R, Kegler M, eds. Emerging theories in health promotion practice and research. San Fransisco: Jossy-Bass 2002:40–70.
20.	Bandura A. Self-efficacy: Towards a unifying theory of behavioural change. *Psychol Rev* 1997;84(2):191–215.
21.	Avery K, Donovan J, Peters TJ, Shaw C, Gotoh M, Abrams P. ICIQ: A brief and robust measure for evaluating the symptoms and impact of urinary incontinence. *Neurourol Urodyn* 2004;23(4):322–330.
22.	Yalcin I, Bump RC. Validation of two global impression questionnaires for incontinence. *Am J Obstet Gynecol* 2003;189:98–101.
23.	Brookes S, Donovan J, Wright M, Jackson S, Abrams P. A scored form of the Bristol Lower Urinary Tract Symptoms questionnaire: data from a randomized controlled trial of surgery for women with stress incontinence. *Am J Obstet Gynecol* 2004;191(1):73–82.
24.	Kelleher C, Cardozo L, Khullar V, Salvatore S. A new questionnaire to assess the quality of life of urinary incontinent women. *BJOG* 1997;104:1374–1379.
25.	Hagen S, Glazener C, Sinclair L, Stark D, Bugge C. Psychometric properties of the Pelvic Organ Prolapse Symptom Score (POP-SS). *BJOG* 2009;116:25–31.
26.	Laycock J. Vaginal examination. In: Laycock J, Haslam J, eds. Therapeutic Management of Incontinence and Pelvic Pain. London: Springer-Verlag 2008:61–63.
27.	Messelink B, Benson T, Berghmans B, Bø K, Corcos J, Fowler C, Laycock J, Huat-Chye Lim P, van Lunsen R, Lycklama a¤ Nijeholt G, Pemberton J, Wang A, Watier A, Van Kerrebroeck P. Standardization of Terminology of Pelvic Floor Muscle Function and Dysfunction: Report from the Pelvic Floor Clinical Assessment Group of the International Continence Society. *Neurourol Urodyn* 2005;24:374–380.
28.	Chen S-Y. The development and testing of the pelvic floor muscle exercise self-efficacy scale. *J Nurs Res* 2004;12(4):257–265.
29.	The EuroQol Group. EuroQol--a new facility for the measurement of health-related quality of life. *Health Policy* 1990;16(3):199–208.
30.	Klovning A, Avery K, Sandvik H, Hunskaar S. Comparison of Two Questionnaires for Assessing the Severity of Urinary Incontinence: The ICIQ-UI SF versus the Incontinence Severity Index. *Neurourol Urodyn* 2009; 28:411–415.
31.	Sherburn M. Bø K, Galea M. Evaluation of outcome measures for stress urinary incontinence in older women. *Neurourol Urodyn* 2009:28(7);715–716.

For peer review only

AUTHOR STATEMENT

S Hagen directed the protocol development, inputting specifically to the trial design and outcome measurement components; J Hay-Smith, D McClurg, S Dean and J Booth developed the trial interventions, focussing on the behaviour change (JHS, SD) and clinical (DM, JB) components; C Glazener and J Norrie contributed their expertise in clinical trial design, specific to the area of incontinence and complex intervention respectively; M Abdel-fattah, W Agur and K Guerrero contributed their research and clinical expertise relating to urinary incontinence to inform the protocol development and its implementation; C Bugge led the development of the process evaluation and qualitative longitudinal case study supported by SD and JHS, ensuring that the longitudinal qualitative study and process evaluation protocol was congruent with and complementary to the trial protocol; A McDonald and G McPherson contributed to the development of the trial management and trial database processes respectively; M Kilonzo developed the health economic methods within the protocol; A Elders developed the statistical methods within the protocol; L Wilson contributed a consumer's perspective to the trial protocol as a whole; S Stratton and N Sergenson

For peer review only - http://bmjopen.bmj.com/site/about/guidelines.xhtml

contributed to the design of the implementation processes for trial management and data collection and management respectively; A Grant contributed expertise in process evaluation to further develop those elements of the protocol.

FUNDING ACKNOWLEDGEMENT

This project, Optimising Pelvic floor Exercises to Achieve Long-term benefits (OPAL) is funded by the NIHR Health Technology Assessment Programme (project reference: 11/71/03). The authors also acknowledge the support of the National Institute for Health Research, through the Comprehensive Clinical Research Network. SD's position is partly supported by the National Institute for Health Research (NIHR) Collaboration for Leadership in Applied Health Research and Care South West Peninsula at the Royal Devon and Exeter NHS Foundation Trust.

DEPARTMENT OF HEALTH AND SOCIAL CARE DISCLAIMER

The views expressed are those of the authors and not necessarily those of the NHS, the NIHR or the Department of Health and Social Care.

DATA STATEMENT

Technical appendix, statistical code, and datasets (subject to agreement with the TSC and funders) will be made available at the end of the trial.

CONFLICTS OF INTERESTS

None of the authors has expressed any competing interests

For peer review only

For peer review only

For peer review only - http://bmjopen.bmj.com/site/about/guidelines.xhtml

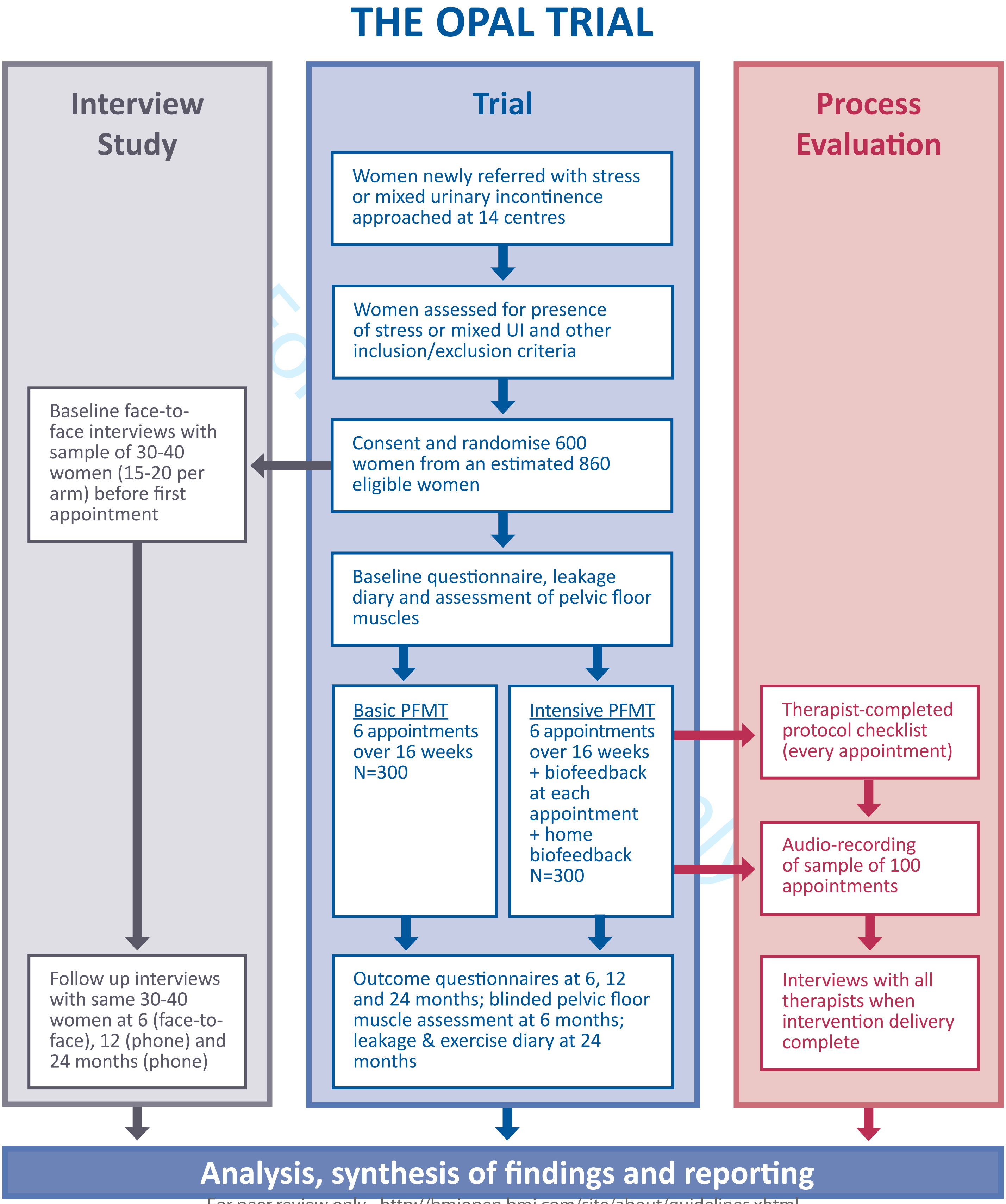
THE OPAL TRIAL
Interview Study
Trial
Process Evaluation
Women newly referred with stress or mixed urinary incontinence approached at 14 centres
Women assessed for presence of stress or mixed UI and other inclusion/exclusion criteria
Baseline face-to-face interviews with sample of 30-40 women (15-20 per arm) before first appointment
Consent and randomise 600 women from an estimated 860 eligible women
Baseline questionnaire, leakage diary and assessment of pelvic floor muscles
Basic PFMT 6 appointments over 16 weeks N=300
Intensive PFMT 6 appointments over 16 weeks + biofeedback at each appointment + home biofeedback N=300
Therapist-completed protocol checklist (every appointment)
Audio-recording of sample of 100 appointments
Follow up interviews with same 30-40 women at 6 (face-to-face), 12 (phone) and 24 months (phone)
Outcome questionnaires at 6, 12 and 24 months; blinded pelvic floor muscle assessment at 6 months; leakage & exercise diary at 24 months
Interviews with all therapists when intervention delivery complete
Analysis, synthesis of findings and reporting

SPIRIT checklist and figure

SPIRIT 2013 Checklist: Recommended items to address in a clinical trial protocol and related documents*

Section/item	Item No	Description
**Administrative information**		
Title	1	Multicentre randomised trial of the effectiveness and cost-effectiveness of basic versus biofeedback-mediated intensive pelvic floor muscle training for female stress or mixed urinary incontinence, OPAL.
Trial registration	2a	Trial identifier and registry name. If not yet registered, name of intended registry http://www.controlled trials.com/ **ISRCTN57746448** on the 10/01/2014
	2b	All items from the World Health Organization Trial Registration Data Set
Protocol version	3	V6 01.12.16
Funding	4	Sources and types of financial, material, and other support. The trial is funded by the United Kingdom National Health Service through the National Institute for Health Research Health Technology Assessment programme, open call Project number **HTA 11/71/03**

For peer review only

For peer review only - http://bmjopen.bmj.com/site/about/guidelines.xhtml

Roles and responsibilities	5a	Suzanne Hagen, PhD – Chief Investigator<br>Doreen McClurg, PhD –urinary incontinence and trials expertise<br>Carol Bugge, PhD –qualitative lead<br>Jean Hay-Smith, PhD- qualitative, behavioural change expert<br>Sarah Dean, PhD- qualitative, behavioural change expert<br>Andy Elders, MSc – statistical and trials expertise<br>Cathryn Glazener, PhD – clinical trials, incontinence expertise<br>Mohamed Abdel-fattah, MRCOG, MB BCh, Consultant Gynaecologist<br>Wael Agur, MRCOG, MB BCh-Consultant Gynaecologist, local PI<br>Jo Booth, PhD-urinary incontinence and nursing expertise<br>Karen Guerrero, MRCOG, MB BCh -Consultant Gynaecologist, local PI<br>John Norrie, MSc – statistical and trials expertise<br>Mary Kilonzo, MSc -health economics<br>Gladys McPherson, PhD - trial randomisation/database expertise<br>Alison McDonald, MSc-trial management expertise<br>Susan Stratton, BSc – Trial Manager<br>Nicole Sergenson, BSc - Data Manager<br>Aileen Grant, PhD- qualitative researcher<br>Lyndsay Wilson - PPI representative
	5b	Name and contact information for the trial sponsor-<br>Professor Jim Woodburn, Glasgow Caledonian University, Glasgow G4 0BA
	5c	Role of study sponsor and funders, if any, in study design; collection, management, analysis, and interpretation of data; writing of the report; and the decision to submit the report for publication, including whether they will have ultimate authority over any of these activities<br><br>The funders reviewed the application and the submission and approved it.

For peer review only

5d	Composition, roles, and responsibilities of the coordinati	ng centre, steering committee, endpoint adjudication committee, data management team, and other individuals or groups overseeing the trial, if applicable (see Item 21a for data monitoring committee)<br><br>The role of the TSC is to provide overall supervision for a trial on behalf of the Trial Sponsor and Trial Funder and to ensure that the trial is conducted to the rigorous standards set out in the Department of Health's Research Governance Framework for Health and Social Care and the Guidelines for Good Clinical Practice. Independent members are: Mr Chris Mayne (Chair), Dr Thomas Chadwick, Mrs Teresa Cook, Veronica Haggar, Ms Kirsty Craig.<br><br>An independent DMEC will review confidential interim analyses of accumulating data annually. The DMEC comprises experts in the clinical field and an expert trial statistician, and will monitor data and make recommendations to the TSC if there are any ethical or safety issues. Dr Steff Lewis (Chair), Ms Sue Hallam, Mr Simon Emery.

	STUDY PERIOD								
	Enrolment	Allocation	Post-Allocation				Close-out	Follow u	
**TIMEPOINT****	*-t1 week-1*	*t1 week 0*	*t2 week 1*	*t3 week 3*	*t4 week 6*	*t5 week 10*	*t6 week 15*	*t7 month 6*	*t8 month 12*
**ENROLMENT:**									
**Eligibility screen**	X								
**Informed consent**	X								
**Allocation**		Randomisation							
**INTERVENTIONS:**									
***(Intervention A)* PFMT intervention**		Shown PFMT exercises. Allocated individualised program		PFMT exercise program					
***(Intervention B)* Biofeedback-mediated PFMT intervention**		Shown PFMT exercises and biofeedback machine demonstration		PFMT exercise program and biofeedback					
**ASSESSMENTS:**									
**Bladder Diary**	X	X							

For peer review only

**PFMT Diary**			X	X	X	X			
**PFMT Diary and Biofeedback diary**			X	X	X	X			
**Blinded Pelvic floor examination**							X		
***Primary outcome* ICIQ-UI SF**	X						X	X	X
Other outcomes	X						X	X	X
**Adverse Events**		X	X	X	X	X			
**PFMT Maintenance recommendations**						X			

*Recommended content can be displayed using various schematic formats. See SPIRIT 2013 Explanation and Elaboration for examples from protocols.
**List specific timepoints in this row.

	6a	Accidental urine leakage (incontinence) is a distressing problem that affects about 1 in 3 women, and the NHS spends large amounts of money treating it. Many women have stress incontinence; that is, accidental urine leakage that happens with coughing, sneezing and physical activity. Based on past research, current UK guidelines recommend that these women are offered at least three months of pelvic floor muscle exercises. The exercises are taught by a specialist physiotherapist or nurse. There is evidence that these exercises can strengthen the muscles and decrease leakage. It is not clear how "intensively" women have to exercise to get a good result that lasts thus improving the woman's quality of life and reducing the likelihood of surgery. Many women have stress incontinence; that is, accidental urine leakage that happens with coughing, sneezing and physical activity. Based on past research, current UK guidelines recommend that these women are offered at least three months of pelvic floor muscle exercises. The exercises are taught by a specialist physiotherapist or nurse. There is evidence that these exercises can strengthen the muscles and decrease leakage. It is not clear how "intensively" women have to exercise to get a good result that lasts thus improving the woman's quality of life and reducing the likelihood of surgery. This research aims to find out whether the use of biofeedback can help to improve the results of pelvic floor muscle exercises in the short- and longer-term. Using a probe inserted into the vagina, biofeedback equipment shows women what their pelvic floor muscles do. Because the muscles are "inside" they are "hidden", and biofeedback allows women to "see" the muscles working as they exercise. "Seeing" the muscles working on the biofeedback screen might help with teaching the exercises and encourage women to continue doing their exercises. A graph can be printed out to show changes in the muscle strength over time. The women who receive biofeedback may exercise more because they are more confident about doing the exercises and the changes that the exercises can achieve.
Background and rationale	6b	Explanation for choice of comparators **Explained in paper**
Objectives	7	Specific objectives or hypotheses **Explained in paper**
Trial design	8	Description of trial design including type of trial (eg, parallel group, crossover, factorial, single group), allocation ratio, and framework (eg, superiority, equivalence, non inferiority, exploratory) **Explained in paper**

Methods: Participants, interventions, and outcomes

For peer review only

For peer review only - http://bmjopen.bmj.com/site/about/guidelines.xhtml

Study setting	9	Description of study settings (eg, community clinic, academic hospital) and list of countries where data will be collected. Reference to where list of study sites can be obtained **Explained in paper**
Eligibility criteria	10	Inclusion and exclusion criteria for participants. If applicable, eligibility criteria for study centres and individuals who will perform the interventions (eg, surgeons, psychotherapists) **Explained in paper**
Interventions	11a	Interventions for each group with sufficient detail to allow replication, including how and when they will be administered **Explained in paper**
	11b	Criteria for discontinuing or modifying allocated interventions for a given trial participant (eg, drug dose change in response to harms, participant request, or improving/worsening disease) **Explained in paper**
	11c	Strategies to improve adherence to intervention protocols, and any procedures for monitoring adherence (eg, drug tablet return, laboratory tests) **Explained in paper**
	11d	Relevant concomitant care and interventions that are permitted or prohibited during the trial **Explained in paper**
Outcomes	12	Primary, secondary, and other outcomes, including the specific measurement variable (eg, systolic blood pressure), analysis metric (eg, change from baseline, final value, time to event), method of aggregation (eg, median, proportion), and time point for each outcome. Explanation of the clinical relevance of chosen efficacy and harm outcomes is strongly recommended **Explained in paper**
Participant timeline	13	Time schedule of enrolment, interventions (including any run-ins and washouts), assessments, and visits for participants. A schematic diagram is highly recommended (see Figure) **Explained in paper**
Sample size	14	Estimated number of participants needed to achieve study objectives and how it was determined, including clinical and statistical assumptions supporting any sample size calculations **Explained in paper**
Recruitment	15	Strategies for achieving adequate participant enrolment to reach target sample size **Explained in paper**

Methods: Assignment of interventions (for controlled trials)

Allocation:

Sequence generation	16a	Method of generating the allocation sequence (eg, computer-generated random numbers), and list of any factors for stratification. To reduce predictability of a random sequence, details of any planned restriction (eg, blocking) should be provided in a separate document that is unavailable to those who enrol participants or assign interventions **Explained in paper**

For peer review only

Allocation concealment mechanism	16b	Mechanism of implementing the allocation sequence (eg, central telephone; sequentially numbered, opaque, sealed envelopes), describing any steps to conceal the sequence until interventions are assigned **Explained in paper**
Implementation	16c	Who will generate the allocation sequence, who will enrol participants, and who will assign participants to interventions **Explained in paper**
Blinding (masking)	17a	Who will be blinded after assignment to interventions (eg, trial participants, care providers, outcome assessors, data analysts), and how **Explained in paper**
	17b	If blinded, circumstances under which unblinding is permissible, and procedure for revealing a participant's allocated intervention during the trial **Explained in paper**
**Methods: Data collection, management, and analysis**		
Data collection methods	18a	Plans for assessment and collection of outcome, baseline, and other trial data, including any related processes to promote data quality (eg, duplicate measurements, training of assessors) and a description of study instruments (eg, questionnaires, laboratory tests) along with their reliability and validity, if known. Reference to where data collection forms can be found, if not in the protocol **Explained in paper**
	18b	Plans to promote participant retention and complete follow-up, including list of any outcome data to be collected for participants who discontinue or deviate from intervention protocols **Explained in paper**
Data management	19	Plans for data entry, coding, security, and storage, including any related processes to promote data quality (eg, double data entry; range checks for data values). Reference to where details of data management procedures can be found, if not in the protocol **Explained in paper**
Statistical methods	20a	Statistical methods for analysing primary and secondary outcomes. Reference to where other details of the statistical analysis plan can be found, if not in the protocol **Explained in paper**
	20b	Methods for any additional analyses (eg, subgroup and adjusted analyses) **Explained in paper**
	20c	Definition of analysis population relating to protocol non-adherence (eg, as randomised analysis), and any statistical methods to handle missing data (eg, multiple imputation) **Explained in paper**
**Methods: Monitoring**		

For peer review only

Data monitoring	21a	Composition of data monitoring committee (DMC); summary of its role and reporting structure; statement of whether it is independent from the sponsor and competing interests; and reference to where further details about its charter can be found, if not in the protocol. Alternatively, an explanation of why a DMC is not needed **Explained in paper**
	21b	Description of any interim analyses and stopping guidelines, including who will have access to these interim results and make the final decision to terminate the trial **Explained in paper**
Harms	22	Plans for collecting, assessing, reporting, and managing solicited and spontaneously reported adverse events and other unintended effects of trial interventions or trial conduct **Explained in paper**
Auditing	23	Frequency and procedures for auditing trial conduct, if any, and whether the process will be independent from investigators and the sponsor **The TSC and DMEC will meet annually during the trial. An independent Chair and members will be appointed by the funder.**

Ethics and dissemination

Research ethics approval	24	Plans for seeking research ethics committee/institutional review board (REC/IRB) approval West of Scotland Research Ethics Committee 4, obtained on the 13th March 2013-13/WS/0048
Protocol amendments	25	Plans for communicating important protocol modifications (eg, changes to eligibility criteria, outcomes, analyses) to relevant parties (eg, investigators, REC/IRBs, trial participants, trial registries, journals, regulators) **Funders, sponsors and R&D will be notified routinely and appropriate approvals gained**
Consent or assent	26a	Who will obtain informed consent or assent from potential trial participants or authorised surrogates, and how (see Item 32) **Explained in paper**
	26b	Additional consent provisions for collection and use of participant data and biological specimens in ancillary studies, if applicable **N/A**
Confidentiality	27	How personal information about potential and enrolled participants will be collected, shared, and maintained in order to protect confidentiality before, during, and after the trial **Explained in paper**
Declaration of interests	28	Financial and other competing interests for principal investigators for the overall trial and each study site **None declared**

For peer review only

Access to data	29	Statement of who will have access to the final trial dataset, and disclosure of contractual agreements that limit such access for investigators **Explained in paper**
Ancillary and post-trial care	30	Provisions, if any, for ancillary and post-trial care, and for compensation to those who suffer harm from trial participation **N/A**
Dissemination policy	31a	Plans for investigators and sponsor to communicate trial results to participants, healthcare professionals, the public, and other relevant groups (eg, via publication, reporting in results databases, or other data sharing arrangements), including any publication restrictions **Explained in paper**
	31b	Authorship eligibility guidelines and any intended use of professional writers **Explained in paper**
	31c	Plans, if any, for granting public access to the full protocol, participant-level dataset, and statistical code **To be discusses and agreed by the PMG and TSC**
**Appendices**		
Informed consent materials	32	Model consent form and other related documentation given to participants and authorised surrogates **Attached as an appendix**
Biological specimens	33	Plans for collection, laboratory evaluation, and storage of biological specimens for genetic or molecular analysis in the current trial and for future use in ancillary studies, if applicable **N/A**

For peer review only

*It is strongly recommended that this checklist be read in conjunction with the SPIRIT 2013 Explanation & Elaboration for important clarification on the items. Amendments to the protocol should be tracked and dated. The SPIRIT checklist is copyrighted by the SPIRIT Group under the Creative Commons "Attribution-NonCommercial-NoDerivs 3.0 Unported" license.

For peer review only

BMJ Open

Effectiveness and cost-effectiveness of basic versus biofeedback-mediated intensive pelvic floor muscle training for female stress or mixed urinary incontinence: protocol for the OPAL randomised trial

Journal:	*BMJ Open*
Manuscript ID	bmjopen-2018-024153.R1
Article Type:	Protocol
Date Submitted by the Author:	19-Sep-2018

Complete List of Authors:	Hagen, Suzanne; Glasgow Caledonian University<br>McClurg, Doreen; Glasgow Caledonian University, NMAHP RU<br>Bugge, Carol; University of Stirling, School of Health Sciences and Sport<br>Hay-smith, Jean; University of Otago Wellington, Rehabilitation Teaching and Research Unit<br>Dean, Sarah; PenCLAHRC University of Exeter Medical School,<br>Elders, A; Glasgow Caledonian University School of Health and Life Sciences, NMAHP Research Unit ; Glasgow Caledonian University<br>Glazener, Cathryn; University of Aberdeen, Health Services Research Unit<br>Abdel-fattah, Mohamed; University of Aberdeen, Obstetrics and Gynaecology<br>Agur, Wael; University of Glasgow, School of Medicine<br>Booth, Jo; Glasgow Caledonian University<br>Guerrero, Karen; NHS Greater Glasgow and Clyde<br>Norrie, John; Edinburgh Clinical Trials Unit, University of Edinburgh No. 9, Bioquarter<br>Kilonzo, M; University of Aberdeen<br>McPherson, Gladys; University of Aberdeen Institute of Applied Health Sciences<br>McDonald, Alison; University of Aberdeen, Health Services Research Unit<br>Stratton, Susan; Glasgow Caledonian University<br>Sergenson, Nicole; Glasgow Caledonian University<br>Grant, Aileen; Robert Gordon University, School of Nursing and Midwifery<br>Wilson, Lyndsay

**Primary Subject Heading**:	Urology
Secondary Subject Heading:	Evidence based practice, Health services research, Obstetrics and gynaecology
Keywords:	urinary incontinence, pelvic floor muscle training, biofeedback, electromyography, adherence, long-term

Title page

Effectiveness and cost-effectiveness of basic versus biofeedback-mediated intensive pelvic floor muscle training for female stress or mixed urinary incontinence: protocol for the OPAL randomised trial

- Corresponding author.

Professor Suzanne Hagen
Nursing, Midwifery and Allied Health Professions Research Unit Glasgow
Caledonian University
Cowcaddens Road, Glasgow G4 0BA
0141 331 8104
s.hagen@gcu.ac.uk

- Co-authors.

Doreen McClurg
Nursing, Midwifery and Allied Health Professions Research Unit,
Glasgow Caledonian University,
Glasgow, UK

Carol Bugge
Faculty of Health Sciences and Sport,
University of Stirling,
Stirling, UK

Jean Hay-Smith
Department of Medicine,
University of Otago,
Dunedin, New Zealand

Sarah Dean
University of Exeter Medical School,
Exeter, UK

Andrew Elders
Nursing, Midwifery and Allied Health Professions Research Unit,
Glasgow Caledonian University,
Glasgow, UK

Cathryn Glazener
Health Services Research Unit,
University of Aberdeen,
Aberdeen, UK

Mohamed Abdel-fattah
Division of Applied Health Sciences,
University of Aberdeen,
Aberdeen, UK

Wael Agur

For peer review only - http://bmjopen.bmj.com/site/about/guidelines.xhtml

NHS Ayrshire and Arran University Hospital,
Crosshouse Hospital,
Kilmarnock, UK

Jo Booth
School of Health and Life Sciences,
Glasgow Caledonian University,
Glasgow, UK

Karen Guerrero
NHS Greater Glasgow and Clyde,
Queen Elizabeth Hospital,
Glasgow, UK

John Norrie
Edinburgh Clinical Trials Unit,
University of Edinburgh,
Edinburgh, UK

Mary Kilonzo
Health Economics Research Unit,
University of Aberdeen,
Aberdeen, UK

Gladys McPherson
The Centre for Healthcare Randomised Trials,
Health Services Research Unit,
University of Aberdeen,
Aberdeen, UK

Alison McDonald
The Centre for Healthcare Randomised Trials,
Health Services Research Unit,
University of Aberdeen,
Aberdeen, UK

Susan Stratton
Nursing, Midwifery and Allied Health Professions Research Unit,
Glasgow Caledonian University,
Glasgow, UK

Nicole Sergenson
Nursing, Midwifery and Allied Health Professions Research Unit,
Glasgow Caledonian University,
Glasgow, UK

Aileen Grant
School of Nursing and Midwifery,
Robert Gordon University,

For peer review only

Aberdeen. UK

Lyndsay Wilson,
C/O Glasgow Caledonian
University
Glasgow, UK

WORD COUNT: 3327

KEYWORDS

Urinary incontinence; pelvic floor muscle training; biofeedback; electromyography; adherence; long-term

For peer review only

For peer review only - http://bmjopen.bmj.com/site/about/guidelines.xhtml

Effectiveness and cost-effectiveness of basic versus biofeedback-mediated intensive pelvic floor muscle training for female stress or mixed urinary incontinence: protocol for the OPAL randomised trial

Suzanne Hagen, Doreen McClurg, Carol Bugge, Jean Hay-Smith, Sarah Dean, Andrew Elders, Cathryn Glazener, Mohamed Abdel-fattah, Wael Agur, Jo Booth, Karen Guerrero, John Norrie, Mary Kilonzo, Gladys McPherson, Alison McDonald, Susan Stratton, Nicole Sergenson, Aileen Grant, Lyndsay Wilson

ABSTRACT

Introduction: Accidental urine leakage is a distressing problem that affects around one in three women. The main types of urinary incontinence (urinary incontinence) are stress, urgency and mixed, with stress being most common. Current UK guidelines recommend that women with UI are offered at least three months of pelvic floor muscle training (PFMT). There is evidence that PFMT is effective in treating UI, however it is not clear how intensively women have to exercise to give the maximum sustained improvement in symptoms, and how we enable women to achieve this. Biofeedback is an adjunct to PFMT that may help women exercise more intensively for longer, and thus may improve continence outcomes when compared to PFMT alone. A Cochrane review was inconclusive about the benefit of biofeedback, indicating the need for further evidence.

Methods and analysis: This multicentre randomised controlled trial will compare the effectiveness and cost-effectiveness of PFMT versus biofeedback-mediated PFMT for women with stress UI or mixed UI. The primary outcome is UI severity at 24 months after randomisation. The primary economic outcome measure is incremental cost per quality-adjusted life-year at 24 months. Six hundred women from UK community, outpatient and primary care settings will be randomised and followed up via questionnaires, diaries and pelvic floor assessment. All participants are offered six PFMT appointments over 16 weeks. The use of clinic and home biofeedback is added to PFMT for participants in the biofeedback group. Group allocation could not be masked from participants and healthcare staff. An intention-to-treat analysis of the primary outcome will estimate the mean difference between the trial groups at 24 months using a general linear mixed model adjusting for minimisation covariates and other important prognostic covariates, including the baseline score.

Ethics and dissemination: Approval granted by the West of Scotland Research Ethics Committee 4 (16/LO/0990). Written informed consent will be obtained from participants by the local research team. Serious adverse events will be reported to the data monitoring and ethics committee, the ethics committee and trial centres as required. A SPIRIT checklist and figure are available for this protocol. The results will be published in international journals and included in the relevant Cochrane Review.

Registration: ISRCTN 57746448.

Keywords: urinary incontinence; pelvic floor muscle training; biofeedback; electromyography; adherence; long-term

STRENGTHS AND LIMITATIONS

1. The intervention being evaluated is highly relevant to UK NHS practice.
2. The primary outcome measure is woman-centred, focusing on the severity of their urine leakage symptoms and the impact on their quality of life.
3. An extensive longitudinal qualitative case study and process evaluation will provide rich data about women's experiences of the intervention.
4. All trial participants receive a high standard of pelvic floor muscle training, therefore the effect of adding biofeedback will need to be large to show between-group differences.
5. Women randomised to the pelvic floor muscle training group could also access biofeedback, as devices are available to buy.

INTRODUCTION

For peer review only

For peer review only - http://bmjopen.bmj.com/site/about/guidelines.xhtml

Urinary incontinence (UI), defined as any involuntary loss of urine,[1] is a common condition in women. The prevalence of UI depends on the definition: using a broad definition, a range of between 5% and 69% is reported, with most studies in the range 25% to 45%.[1] The main types of UI are stress, urgency and mixed, with stress being most prevalent (accounts for around half of all UI), followed by mixed UI (stress and urgency combined) (most studies report 7.5-25%), and fewer having urgency UI alone (most studies report 1-7%). The cost to the UK NHS annually of treating clinically significant UI in women was estimated in the Leicestershire MRC Incontinence Study[2] as £233M, not including the personal costs borne by the women which were estimated to be £178M in the same study.[3] UI significantly impacts on daily living for the majority of women, with an associated increased prevalence of major depression.[4] Among older women, social isolation, psychological distress[5] and increased risk of admission to long-term care institutions[6] feature highly. Thus UI is prevalent and costly to women, the NHS and society both financially and in terms of physical and mental wellbeing.

Currently, supervised pelvic floor muscle training (PFMT) of at least three months' duration is the first-line treatment for stress and mixed UI.[7] PFMT involves the regular practice of repeated voluntary pelvic floor muscle contractions, with sufficient exercise progression, in order to produce a training effect on the muscles. The aim of a PFMT programme is to increase the strength of the muscles, to build up muscle volume and thus improve structural support; to increase contraction endurance; to improve muscle resting tone; to improve muscle recruitment through improved nerve function and properties of muscle fibres; and improve cognitive awareness of body posture and a relaxed versus an un-relaxed state of the pelvic floor. In addition, a PFMT programme involves instruction on contracting and relaxing the pelvic floor muscles to improve coordination and functional use (termed the 'Knack') to occlude the urethra during physical activities that increase abdominal pressure and precipitate UI. Pelvic floor muscle contraction can also be used to inhibit a bladder contraction at the time of urgency as a method of urge suppression.

To produce improvements in muscle strength and endurance the basic physiological principles must be adhered to:[8] overload (muscles need to perform more work than usual to the point of fatigue); specificity (muscles must be trained with exercise or physical activity that replicates as closely as possible the functional movement required); maintenance and reversibility (benefits of the exercise will reverse if they are not undertaken on a regular basis). There is evidence to suggest that for effective resistance training in skeletal muscles in adults, two to four sets of 8 to 12 slow to moderate velocity, moderate to maximal intensity contractions be performed per day, 2 to 3 days per week with progression over 16 weeks.[9,10]

Biofeedback is commonly used as an adjunct to PFMT. Biofeedback is the technique by which information about a normally unconscious physiological process is presented to the patient and/or the therapist as a visual, auditory or tactile signal.[11] Electromyography (EMG) is the study of minute electrical potentials produced by depolarisation of muscle membrane.[12] In EMG biofeedback, electrical activity arising from muscle activity (during exercise and voluntary effort) is recorded in microvolts and displayed as a visual or auditory signal for both patient and therapist to view. Within a PFMT programme, an internal vaginal probe is used to record electrical information from pelvic floor muscles through surface recordings. The probe is connected by cables to a biofeedback unit. Handheld units with a small visual display screen are available for home use. The display provides a visual representation of the muscles contracting and relaxing, allowing monitoring of strength, endurance and repetitions. The effectiveness of using biofeedback to augment PFMT for UI in women has been the subject of a Cochrane review, the conclusions of which were that women who had PFMT including biofeedback were more likely to report cure or improvement of UI than those who had PFMT without biofeedback, but this finding was potentially confounded by more health professional contact and supervised treatment time in the biofeedback groups.[13] Biofeedback in general is considered an evidence-based Behaviour Change Technique (BCT) according to the BCT Taxonomy.[14]

The effect of PFMT relies firstly on sufficient exercise being undertaken for a long enough period to strengthen and

For peer review only - http://bmjopen.bmj.com/site/about/guidelines.xhtml

hypertrophy the muscles, and secondly the continuation of sufficient exercise so that strength is maintained.[15] Thus to intensify PFMT, adherence must be maximised both initially (i.e. uptake or adoption of the exercise programme) and then continued over the longer-term (maintenance). Based on current evidence, it is not clear if the apparent benefit of biofeedback can be attributed to the biofeedback or to some other variable such as more health professional contact in those women receiving biofeedback.[13,16] A common problem in previous trials was the failure to clearly state the purpose of biofeedback, or to describe the intervention protocol.[13] Thus, it was not clear if the way the biofeedback was used could theoretically or in practice qualitatively change the intensity or effectiveness of the PFMT. In our trial the purpose of biofeedback is twofold: to enhance uptake initially as women will know they are performing the correct technique, and to support ongoing exercise training adherence over the longer-term.

The aim of this trial is to determine the effectiveness and cost-effectiveness of PFMT compared to biofeedback-mediated PFMT for the treatment of stress or mixed UI. Both trial groups will have the same PFMT programme and same amount of health professional contact to establish if the addition of biofeedback improves incontinence outcomes.

METHODS AND ANALYSIS

This trial design is a parallel group multicentre randomised controlled trial, comparing effectiveness and cost-effectiveness of PFMT versus biofeedback-mediated PFMT for women with stress or mixed UI. It is set in UK community, outpatient and primary care settings, where continence care is usually provided. The trial includes a nested longitudinal qualitative case study and process evaluation (Figure 1 OPAL study figure). The trial is assessing superiority of PFMT plus biofeedback over PFMT alone. The start date is 1 September 2013 and finish date 30 November 2018 (Figure 2 OPAL DRAFT SPIRIT-BMJ Open resubmission August 2018).

Trial groups

Participants in this trial will be randomly assigned to either PFMT or biofeedback-mediated PFMT. Allocation will be generated by a web-based programme developed and implemented by The Centre for Healthcare Randomised Trials, situated remotely from the trial office and recruiting centres.

Prior to randomisation, as part of the screening process, a visual and digital assessment of the woman's vagina and pelvic floor muscles will be carried out to provide accurate knowledge of the condition of the perineum and vagina (including areas of pain or increased tone), and the woman's capacity for contracting and relaxing her pelvic floor muscles.

Control group (PFMT)

After randomisation, women will be taught by a trained therapist (nurse or physiotherapist) the correct exercise technique and this will be confirmed on digital palpation with the woman in the supine position. Women will be encouraged to become aware of the sensation or feeling of contracting and holding the appropriate muscles, and also of relaxing them.

Women in the PFMT group will be offered six appointments with the trained therapist over a 16-week period at weeks 0, 1, 3, 6, 10 and 15. During these appointments the PFMT programme will be delivered and home exercise prescribed.

The initial PFMT programme will be identified and agreed between the individual woman and her therapist over the first and second appointment, according to the woman's ability. The exercise programme will be practised during the appointments to allow the therapist to assess progress and adjust the programme as necessary. Home exercise will be prescribed after the first appointment, or as soon as the therapist confirms a correct technique has

For peer review only - http://bmjopen.bmj.com/site/about/guidelines.xhtml

been achieved.

There are two main components to the PFMT protocol: resistance training and counterbracing (also called 'The Knack'[17]). Initial exercise prescription in terms of number and type of contractions, length of hold, number of sets, position, will be informed by the PERFECT assessment.[18] The starting dose comprises the number (and duration of hold) of near maximal contractions a woman is able to complete before the pelvic floor muscles reach fatigue. The programme is progressed (e.g. increasing the number of repetitions by one, or duration of hold by one second, each week) according to the woman's ability to reach her individualised goal. For example, a goal might be to exercise 3 days per week. On these days the women could aim for 3 exercise sets, with 3 minutes between sets. Each set could comprise 10 repetitions held for 10 seconds each, with 10 seconds rest between, followed by 10 fast contractions. Counterbracing is taught with a cough – a pre-contraction of the pelvic floor muscles to be held until the abdominal/diaphragmatic contraction ceases - and over time progressed to include other functional activities as clinically indicated. BCTs are built in to the PFMT protocol. The main categories of BCTs included are: beliefs, emotions and information; teaching and confirming PFM contraction, practising skills; goal setting; action planning; problem solving. A set of 30 core BCTs are to be used if possible at each appointment (e.g. agree and record PFMT action plan in new exercise diary) and a further set of 17 optional BCTs are to be used at the therapist's discretion (e.g. discuss pros and cons of doing PFMT). The use of the BCTs is recorded by therapists in a checklist.

No biofeedback equipment is used in the control group, although verbal feedback based on digital vaginal palpation is permitted.

Experimental group (PFMT plus EMG biofeedback)

The use of EMG biofeedback is included in the experimental group in addition to all that is described above for the control group (both PFMT and BCT protocols). Hand-held EMG biofeedback devices with Bluetooth technology, which measure and monitor pelvic floor muscle contractions, are used to deliver biofeedback. The same type of device is used throughout the trial, both in clinic and at home.

Participants randomised to receive PFMT plus biofeedback will have a biofeedback protocol incorporated into their PFMT appointments, and will be given a biofeedback unit to use during home exercise sessions between appointments. The content of the biofeedback protocol is underpinned by the Information-Motivation-Behavioral Skills (IMB) model of behaviour change,[19] incorporating Social Cognitive Theory,[20] an evidence-based theory relating to self-efficacy and perseverance. It is hypothesised that self-efficacy for PFMT is enhanced because biofeedback offers information to women about a normally 'hidden' muscle activity. Biofeedback also supports motivation through providing audio and visual feedback (graphs on screen which can be printed off) on the 'effort/performance' of the muscle contraction, which facilitates tracking changes in muscle strength and performance. In turn this enhances behavioural skills through improving the performance of a muscle contraction, and the timing of a contraction, to reduce leakage with increases in intra-abdominal pressure (e.g. during cough, sneeze, lift). In this way the biofeedback 'intensive' trial group incorporates behaviour change techniques additional to those in the control group, that aim to enhance self-efficacy.

Training in intervention delivery

All therapists delivering the trial interventions are clinical specialists or advanced practitioners already working in the area of continence and will receive training from the trial team on using the biofeedback units and associated software, implementing the PFMT, BCT and biofeedback protocols and completing the trial paperwork to ensure standardisation across centres. An Intervention Training Manual will be provided to each centre covering details of the intervention development and delivery for both the control and experimental groups. Practical sessions using the biofeedback units will be incorporated into the training and there will be a forum for discussion with opportunities for questions. All centres will use the same model of biofeedback unit for clinic and home use.

For peer review only - http://bmjopen.bmj.com/site/about/guidelines.xhtml

Data collection

Data will be collected via participant-completed questionnaires at baseline, 6, 12 and 24 months. In addition, at baseline women will record their leakage episodes in a 3-day bladder diary. During the 16-week intervention women will complete a pelvic floor muscle exercise diary. At 6 months, women have a blinded assessment of their pelvic floor muscle function to quantify pelvic floor muscle function.

Regarding the data collection tools, the ICIQ-UI SF, the ICIQ-FLUTS, the ICIQ-LUTSqol, and the POP-SS are instruments with evidence of reliability, validity and responsiveness. The PGI-I, the Self-Efficacy for PFMT scale and the EQ-5D-3L have evidence of reliability and validity. The ICIQ Bowel Short Form used is an instrument not fully validated, but used because of its brevity and the lack of an alternative validated short bowel symptom questionnaire. The Oxford scale and ICS method of assessing pelvic floor function are standardised methods of digitally assessing the pelvic floor recognised by the International Continence Society. The remaining data are gathered via individual item questions focused on gathering specific information, e.g. relating to whether a participant has received any other treatment for UI, and what incontinence products they have used.

Primary outcome measure: The severity of UI at 24 months is the primary outcome, as measured by the validated International Consultation on Incontinence Questionnaire - Urinary Incontinence Short Form (ICIQ-UI SF) which encompasses urinary leakage frequency, amount and interference with everyday life (total score 0-21, higher score indicates more severe UI).[21]

Secondary outcomes measures:

- Perceived improvement in UI (PGI-I)[22]
- Number with UI cured/improved (derived from the ICIQ-UI SF)[21]
- Uptake of surgery for UI
- Uptake of other treatment for UI
- Other lower urinary tract symptoms (e.g. urgency, nocturia, pain, frequency) (ICIQ-FLUTS)[23]
- UI-specific quality of life (ICIQ-LUTSqol)[24]
- Prolapse symptoms (POP-SS)[25]
- Bowel symptoms (early version of ICIQ Bowel Short Form)
- Pelvic floor muscle function (Oxford scale,[26] ICS method[27])
- Self-efficacy for PFMT (PFME self-efficacy scale)[28]
- Adherence to prescribed PFMT programme (derived from pelvic floor muscle exercise diary/follow-up questionnaire)
- Cost and use of NHS services
- Cost to the women and their families/carers
- Incremental cost per quality-adjusted life-year (EQ-5D-3L)[29]

Centre Selection

This trial will recruit from community, primary and secondary care settings across the UK. The first three centres will be identified and will start recruiting, providing an internal pilot. Experience of recruitment and data generated from these centres over three months will be used to fine-tune trial processes and protocols at new centres. Recruitment at other centres will be staggered with potentially more than 20 centres recruiting to the trial. Potential centres complete a feasibility questionnaire asking about: the number of women they could recruit and how they would be identified; available staff, facilities and time to commit to the trial; previous experience of undertaking trials; any challenges and risks anticipated. A selection will be made on this basis.

For peer review only - http://bmjopen.bmj.com/site/about/guidelines.xhtml

Participant Selection

Women attending for a first continence appointment, or for a first outpatient appointment where UI is the presenting complaint, will be identified in advance from clinic lists in each centre, over the trial recruitment period. An OPAL Participant Information Leaflet will be given to potentially eligible women either before or at their routine appointment. When a woman attends for her appointment with her continence specialist they will confirm a clinical diagnosis of stress UI or mixed UI (those with urgency UI alone will be excluded), and assess other trial eligibility criteria by undertaking a vaginal examination as part of their routine care. If a woman is eligible and willing to take part, informed consent will be obtained by a member of the local research team.

Inclusion criteria

- Women 18 years of age or above, presenting with a new episode of stress or mixed UI, who are willing and suitable to be randomised.

Exclusion criteria

- Women who have urgency UI alone;
- Women who have had formal instruction in PFMT in the last year;
- Women who are unable to contract their pelvic floor muscles;
- Women who are pregnant or are less than 6 months postnatal;
- Women who have prolapse greater than stage II (>1cm below the hymen on Valsalva);
- Women who are currently having treatment for pelvic cancer;
- Women who have cognitive impairment affecting capacity to give informed consent;
- Women who have neurological disease (Multiple Sclerosis, Parkinson's Disease, Stroke, Motor Neurone Disease, Spinal Injury);
- Women with a known nickel allergy or sensitivity;
- Women who are currently participating in other research relating to their UI.

Randomisation

Women who are eligible and willing to take part will be randomised to either the experimental or control group via the automated computer randomisation application developed at the Centre for Healthcare Randomised Trials (CHaRT), University of Aberdeen. The web-based application will be accessed by staff at the centre to determine the participant's group. Allocation will be minimised on: type of UI (stress UI or mixed UI), centre, age (<50/≥50 years) and UI severity at baseline (ICIQ-UI SF score <13/≥ 13).[30]

Masking

Statistical analysis will be conducted by research staff with the group allocation masked, using study identity numbers only to identify women and questionnaires. Due to the nature of the interventions, group allocation cannot be concealed from the women, the therapists delivering the intervention or those entering data. The clinician undertaking 6-month pelvic floor muscle assessments will not be aware of the woman's group allocation.

Data collection and management

Data collection and entry is managed by experienced staff at the OPAL Trial Office situated in NMAHP Research Unit, Glasgow Caledonian University. Data are entered into the CHaRT web-based database. The final trial dataset will be accessed only by the trial statisticians and health economists. Trained therapists or research nurses at centres collect data on paper Case Report Forms at every participant appointment. Centre staff enter the screening and

For peer review only - http://bmjopen.bmj.com/site/about/guidelines.xhtml

eligibility data directly onto the web-based database in order to randomise participants. Participants complete paper diaries throughout treatment and outcome questionnaires at baseline, 6, 12 and 24 months' post randomisation. Paper copies of Case Report Forms, diaries and questionnaires are returned by post to the OPAL Trial Office to be entered onto the database. Copies of Case Report Forms are also securely stored at each centre in accordance with local ethics protocols. Data from the longitudinal qualitative case study and process evaluation are gathered alongside and in addition to the main trial data and are described in the companion paper.

Statistical analysis and sample size

All analyses will be according to the Statistical Analysis Plan and Economic Analysis Plan that will be agreed in advance with the Project Management Group (PMG) and the Trial Steering Committee (TSC). A single main analysis will be performed at the end of the trial when 24-month follow up has been completed. An independent Data Monitoring and Ethics Committee (DMEC) will review confidential interim analyses of accumulating data at its discretion, at least annually. Analysis will be based on the intention-to-treat principle, such that women will be analysed in the groups to which they were allocated regardless of the intervention received.

The main effectiveness analysis will estimate the mean difference in the primary outcome (ICIQ-UI SF score) (and 95% confidence intervals) between the experimental and control groups at 24 months using a general linear mixed model, adjusting for minimisation covariates, therapist type and baseline score. Centre and therapist type will be fitted, where possible, as random effects. Missing outcome data will be estimated using multiple imputation and included in a sensitivity analysis Secondary outcomes will be analysed in a similar manner using an appropriate generalised linear model. Complier average causal effect analysis of the primary outcome will be conducted to investigate the impact of non-compliance. Subgroup analyses by type of incontinence (stress UI or mixed UI), type of therapist (physiotherapist or nurse), participant age (<50/≥50 years) and baseline UI severity (ICIQ-UI SF score <13/≥ 13) are also planned.

The primary economic outcome measure of cost effectiveness is incremental cost per quality-adjusted life-year (QALY) at 24 months undertaken from the NHS perspective. QALYs will be based on responses to the EQ-5D-3L[30] and the ICIQ-LUTSqol.[24] Personal patient resource use data will be incorporated into the analysis to report on a wider perspective. Where appropriate the analysis of incremental costs, effectiveness and cost-effectiveness will be based on similar statistical models as those outlined in the statistical analysis section above. This 'within' trial analysis will include both deterministic and stochastic sensitivity analyses to explore statistical and other forms (e.g. around unit costs or the source of utility estimates) of uncertainty. Similar subgroup analysis will be performed in the economic analysis as defined in the statistical analysis if deemed relevant. If relevant an economic model will be developed to provide additional information for policy makers.

Assuming a clinically meaningful difference of 3 points on the ICIQ-UI SF score (e.g. change from leaking urine "once a day" to "never"), which is similar to the minimal clinically important difference of 2.5 reported in a study of older women,[31] and a conservative estimate for the SD of 10, a sample size of 234 per group would detect this difference (standardised effect size of 0.3) with 90% power at the 5% level of significance (2- sided alpha). Allowing for 22% drop out, we will randomise 300 women per group. With 600 subjects we will also have 90% power to detect important differences in secondary outcomes.

Safety measures

The OPAL trial involves treatments for UI which are well established in clinical practice, therefore adverse effects (although these are unlikely) will be those observed in everyday practice associated with the use of PFMT and biofeedback. In the OPAL trial all serious adverse events experienced by a research participant will be reported to the main research ethics committee if they occur within 30 days of the participant's last PFMT appointment and

For peer review only - http://bmjopen.bmj.com/site/about/guidelines.xhtml

where in the opinion of the Chief Investigator and/or the Chair of the DMEC the event is related and unexpected.

Patient involvement

Women with experience of urinary incontinence are involved in the design, delivery and oversight of the trial through membership on the PMG and TSC. A lay summary of the findings will be sent to participants involved in the trial.

ETHICS AND DISSEMINATION

The trial will be conducted in accordance with the ICH GCP Note for Guidance on Good Clinical Practice. Favourable ethics opinion covering recruitment across all UK NHS centres was obtained from the West of Scotland REC 4 (approved 13th March 2013, reference number 13/WS/0048) and local R&D departments. Trial safety and progress will be overseen by the independent DMEC and the TSC. Meetings of these committees will take place annually.

Final trial results will be disseminated to the funding body, the NIHR Health Technology Assessment Programme. The trial results will be then submitted to peer-reviewed international academic journals and presented at international conferences. Results will also contribute to the relevant Cochrane review. Participants will be provided with a summary of the results.

REFERENCES

1. Abrams P, Cardozo L, Wagg A, Wein A. Incontinence, 6th Edition. Health Publication Ltd 2017.
2. Perry S, Shaw C, Assassa P, et al. An epidemiological study to establish the prevalence of urinary symptoms and felt need in the community: the Leicestershire MRC Incontinence Study. Leicestershire MRC Incontinence Study Team. *Journal of Public Health* 2000;22(3):427–34.
3. Turner DA, Shaw C, McGrother CW, H.M. Dallosso, Cooper NJ. The MRC Incontinence Team. The cost of clinically significant urinary storage symptoms for community dwelling adults in the UK. *BJU Int* 2004;93(9):1246–52.
4. Melville JL, Fan MY, Rau H, Nygaard IE, Katon WJ. Major depression and urinary incontinence in women: temporal associations in an epidemiologic sample. *Am J Obstet Gynecol* 2009;201:490el–490e7.
5. Bognor HR, Gallo JJ, Sammel MD, Ford DE, Armenian HK, Eaton WW. Urinary incontinence and psychological distress in community-dwelling older adults. *J Am Geriatr Soc* 2002;50:489–495.
6. Thom DH, Haan MN, Van Den Eeden SK. Medically recognised urinary incontinence and risks of hospitalisation, nursing home admission and mortality. *Age Ageing* 1997;26:367–374.
7. Welsh A. Urinary incontinence–the management of urinary incontinence in women. National Collaborating Centre for Women's and Children's Health. The National Institute for Health and Clinical Excellence. National Institute for Health and Clinical Excellence, Royal College of Obstetricians and Gynaecologists. London: RCOG Press 2006.
8. McArdle WD, Katch FI. Katch VL. Principles of Training. In Essentials of Exercise Physiology. Philadelphia: Lea and Febiger 1994:346–349.
9. Garber CE, Blissmer B, Deschenes MR, Franklin BA, Lamonte MJ, Lee IM, Nieman DC, Swain DP. American College of Sports Medicine position stand. Quantity and quality of exercise for developing and maintaining cardiorespiratory, musculoskeletal, and neuromotor fitness in apparently healthy adults: guidance for prescribing exercise. *Med Sci Sports Exerc* 2011;43(7):1334–59.
10. Kraemer WJ, Adams K, Cafarelli E, Dudley GA, Dooly C, Feigenbaum MS, Fleck SJ, Franklin B, Fry AC, Hoffman JR, Newton RU. American College of Sports Medicine position stand. Progression models in

For peer review only - http://bmjopen.bmj.com/site/about/guidelines.xhtml

resistance training for healthy adults. *Med Sci Sports Exerc* 2002;34(2):364–80.

11. Abrams P, Cardozo L, Fall M, Griffiths D, Rosier P, Ulmsten U, van Kerrebroeck P, Victor A, Wein A. The standardisation of terminology of lower urinary tract function: Report from the standardisation sub-committee of the International Continence Society. *Neurourol Urodyn* 2002;21(2):167–78.
12. Siroky MB. Electromyograph of the perineal floor. *Urol Clin North Am* 1996;23(2):299–307.
13. Herderschee R, Hay-Smith EJ, Herbison GP, Roovers JP, Heineman MJ. Feedback or biofeedback to augment pelvic floor muscle training for urinary incontinence in women. *Cochrane Libr* 2011 Jan 1.
14. Michie S, Richardson M, Johnston M, Abraham C, Francis J, Hardeman W, Eccles MP, Cane J, Wood CE. The behavior change technique taxonomy (v1) of 93 hierarchically clustered techniques: building an international consensus for the reporting of behavior change interventions. *Ann Behav Med* 2013;46(1):81–95.
15. Bø K. Pelvic floor muscle training is effective in treatment of female stress urinary incontinence, but how does it work? *Int Urogynecol J* 2004;15(2):76–84.
16. Imamura M, Abrams P, Bain C, Buckley B, Cardozo L, Cody J, Cook J, Eustice S, Glazener CM, Grant A, Hay-Smith J. Systematic review and economic modelling of the effectiveness and cost-effectiveness of non-surgical treatments for women with stress urinary incontinence. *Health Technol Assess* 2010.
17. Miller JM, Ashton-Miller JA, DeLancey JO. A pelvic muscle precontraction can reduce cough-related urine loss in selected women with mild SUI. *J Am Geriatr Soc* 1998;46(7):870–4.
18. Laycock J, Jerwood D. Pelvic floor muscle assessment: the PERFECT scheme. *Physiotherapy* 2001;87(12):631–42.
19. Fisher JD, Fisher WA. The information-motivation-behavioral skills model. In: DiClemente R, Crosby R, Kegler M, eds. Emerging theories in health promotion practice and research. San Fransisco: Jossy-Bass 2002:40–70.
20. Bandura A. Self-efficacy: Towards a unifying theory of behavioural change. *Psychol Rev* 1997;84(2):191–215.
21. Avery K, Donovan J, Peters TJ, Shaw C, Gotoh M, Abrams P. ICIQ: A brief and robust measure for evaluating the symptoms and impact of urinary incontinence. *Neurourol Urodyn* 2004;23(4):322–330.
22. Yalcin I, Bump RC. Validation of two global impression questionnaires for incontinence. *Am J Obstet Gynecol* 2003;189:98–101.
23. Brookes S, Donovan J, Wright M, Jackson S, Abrams P. A scored form of the Bristol Lower Urinary Tract Symptoms questionnaire: data from a randomized controlled trial of surgery for women with stress incontinence. *Am J Obstet Gynecol* 2004;191(1):73–82.
24. Kelleher C, Cardozo L, Khullar V, Salvatore S. A new questionnaire to assess the quality of life of urinary incontinent women. *BJOG* 1997;104:1374–1379.
25. Hagen S, Glazener C, Sinclair L, Stark D, Bugge C. Psychometric properties of the Pelvic Organ Prolapse Symptom Score (POP-SS). *BJOG* 2009;116:25–31.
26. Laycock J. Vaginal examination. In: Laycock J, Haslam J, eds. Therapeutic Management of Incontinence and Pelvic Pain. London: Springer-Verlag 2008:61–63.
27. Messelink B, Benson T, Berghmans B, Bø K, Corcos J, Fowler C, Laycock J, Huat-Chye Lim P, van Lunsen R, Lycklama a¤ Nijeholt G, Pemberton J, Wang A, Watier A, Van Kerrebroeck P. Standardization of Terminology of Pelvic Floor Muscle Function and Dysfunction: Report from the Pelvic Floor Clinical Assessment Group of the International Continence Society. *Neurourol Urodyn* 2005;24:374–380.
28. Chen S-Y. The development and testing of the pelvic floor muscle exercise self-efficacy scale. *J Nurs Res* 2004;12(4):257–265.
29. The EuroQol Group. EuroQol--a new facility for the measurement of health-related quality of life. *Health Policy* 1990;16(3):199–208.

For peer review only - http://bmjopen.bmj.com/site/about/guidelines.xhtml

30. Klovning A, Avery K, Sandvik H, Hunskaar S. Comparison of Two Questionnaires for Assessing the Severity of Urinary Incontinence: The ICIQ-UI SF versus the Incontinence Severity Index. *Neurourol Urodyn* 2009; 28:411–415.

31. Sherburn M. Bø K, Galea M. Evaluation of outcome measures for stress urinary incontinence in older women. *Neurourol Urodyn* 2009:28(7);715–716.

AUTHOR STATEMENT

S Hagen directed the protocol development, inputting specifically to the trial design and outcome measurement components; J Hay-Smith, D McClurg, S Dean and J Booth developed the trial interventions, focussing on the behaviour change (JHS, SD) and clinical (DM, JB) components; C Glazener and J Norrie contributed their expertise in clinical trial design, specific to the area of incontinence and complex intervention respectively; M Abdel-fattah, W Agur and K Guerrero contributed their research and clinical expertise relating to urinary incontinence to inform the protocol development and its implementation; C Bugge led the development of the process evaluation and qualitative longitudinal case study supported by SD and JHS, ensuring that the longitudinal qualitative study and process evaluation protocol was congruent with and complementary to the trial protocol; A McDonald and G McPherson contributed to the development of the trial management and trial database processes respectively; M Kilonzo developed the health economic methods within the protocol; A Elders developed the statistical methods within the protocol; L Wilson contributed a consumer's perspective to the trial protocol as a whole; S Stratton and N Sergenson contributed to the design of the implementation processes for trial management and data collection and management respectively; A Grant contributed expertise in process evaluation to further develop those elements of the protocol.

FUNDING

This work is supported by the NIHR Health Technology Assessment Programme (project reference: 11/71/03). The authors also acknowledge the support of the National Institute for Health Research, through the Comprehensive Clinical Research Network. SD's position is partly supported by the National Institute for Health Research (NIHR) Collaboration for Leadership in Applied Health Research and Care South West Peninsula at the Royal Devon and Exeter NHS Foundation Trust. The views expressed are those of the authors and not necessarily those of the NHS, the NIHR or the Department of Health.

DATA STATEMENT

Technical appendix, statistical code, and datasets (subject to agreement with the TSC and funders) will be made available at the end of the trial.

CONFLICTS OF INTERESTS

None of the authors has expressed any competing interests

FIGURE 1: OPAL study figure

FIGURE 2: OPAL DRAFT SPIRIT-BMJ Open resubmission August 2018

For peer review only

For peer review only - http://bmjopen.bmj.com/site/about/guidelines.xhtml

For peer review only

For peer review only - http://bmjopen.bmj.com/site/about/guidelines.xhtml

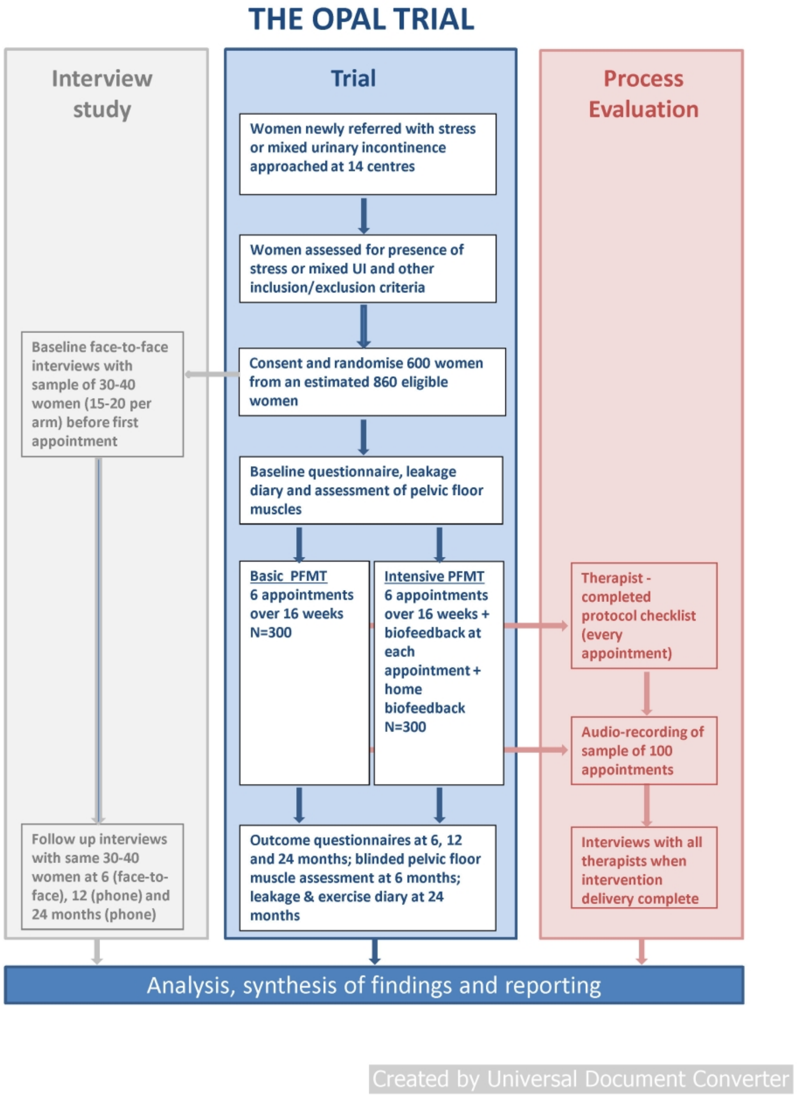

209x297mm (300 x 300 DPI)

		STUDY PERIOD								
	Enrolment	Allocation	Post-Allocation				Close-out	Follow up		
**TIMEPOINT****	*-t1 week-1*	*t1 week 0*	*t2 week 1*	*t3 week 3*	*t4 week 6*	*t5 week 10*	*t6 week 15*	*t7 month 6*	*t8 month 12*	*t9 month 24*
**ENROLMENT:**										
**Eligibility screen**	X									
**Informed consent**	X									
**Allocation**		Randomisation								
**INTERVENTIONS:**										
***(Experimental group)* PFMT intervention**		Taught PFMT exercises.	PFMT exercise program daily							
***(Control group)* Biofeedback-intensified PFMT intervention**		Taught PFMT exercises and biofeedback machine use	PFMT exercise program daily and biofeedback							
**ASSESSMENTS:**										
**Bladder Diary**	X									
**PFMT Diary**			X	X	X	X				

Created by Universal Document Converter

297x209mm (300 x 300 DPI)

SPIRIT checklist and figure

SPIRIT
STANDARD PROTOCOL ITEMS: RECOMMENDATIONS FOR INTERVENTIONAL TRIALS

SPIRIT 2013 Checklist: Recommended items to address in a clinical trial protocol and related documents*

Section/item	Item No	Description
**Administrative information**		
Title	1	Multicentre randomised trial of the effectiveness and cost-effectiveness of basic versus biofeedback-mediated intensive pelvic floor muscle training for female stress or mixed urinary incontinence, OPAL.
Trial registration	2a	Trial identifier and registry name. If not yet registered, name of intended registry http://www.controlled trials.com/ **ISRCTN57746448** on the 10/01/2014
	2b	All items from the World Health Organization Trial Registration Data Set
Protocol version	3	V6 01.12.16
Funding	4	Sources and types of financial, material, and other support. The trial is funded by the United Kingdom National Health Service through the National Institute for Health Research Health Technology Assessment programme, open call Project number **HTA 11/71/03**

For peer review only

For peer review only - http://bmjopen.bmj.com/site/about/guidelines.xhtml

Roles and responsibilities	5a	Suzanne Hagen, PhD – Chief Investigator<br>Doreen McClurg, PhD –urinary incontinence and trials expertise<br>Carol Bugge, PhD –qualitative lead<br>Jean Hay-Smith, PhD- qualitative, behavioural change expert<br>Sarah Dean, PhD- qualitative, behavioural change expert<br>Andy Elders, MSc – statistical and trials expertise<br>Cathryn Glazener, PhD – clinical trials, incontinence expertise<br>Mohamed Abdel-fattah, MRCOG, MB BCh, Consultant Gynaecologist<br>Wael Agur, MRCOG, MB BCh-Consultant Gynaecologist, local PI<br>Jo Booth, PhD-urinary incontinence and nursing expertise<br>Karen Guerrero, MRCOG, MB BCh -Consultant Gynaecologist, local PI<br>John Norrie, MSc – statistical and trials expertise<br>Mary Kilonzo, MSc -health economics<br>Gladys McPherson, PhD - trial randomisation/database expertise<br>Alison McDonald, MSc-trial management expertise<br>Susan Stratton, BSc – Trial Manager<br>Nicole Sergenson, BSc - Data Manager<br>Aileen Grant, PhD- qualitative researcher<br>Lyndsay Wilson - PPI representative
	5b	Name and contact information for the trial sponsor-<br>Professor Jim Woodburn, Glasgow Caledonian University, Glasgow G4 0BA
	5c	Role of study sponsor and funders, if any, in study design; collection, management, analysis, and interpretation of data; writing of the report; and the decision to submit the report for publication, including whether they will have ultimate authority over any of these activities<br><br>The funders reviewed the application and the submission and approved it.

For peer review only

5d	Composition, roles, and responsibilities of the coordinating cent	re, steering committee, endpoint adjudication committee, data management team, and other individuals or groups overseeing the trial, if applicable (see Item 21a for data monitoring committee)<br><br>The role of the TSC is to provide overall supervision for a trial on behalf of the Trial Sponsor and Trial Funder and to ensure that the trial is conducted to the rigorous standards set out in the Department of Health's Research Governance Framework for Health and Social Care and the Guidelines for Good Clinical Practice. Independent members are: Mr Chris Mayne (Chair), Dr Thomas Chadwick, Mrs Teresa Cook, Veronica Haggar, Ms Kirsty Craig.<br><br>An independent DMEC will review confidential interim analyses of accumulating data annually. The DMEC comprises experts in the clinical field and an expert trial statistician, and will monitor data and make recommendations to the TSC if there are any ethical or safety issues. Dr Steff Lewis (Chair), Dr Sue Hallam, Mr Simon Emery.

Background and rationale

For peer review only

6a Accidental urine leakage (incontine		nce) is a distressing problem that affects about 1 in 3 women, and the NHS spends large amounts of money treating it. Many women have stress incontinence; that is, accidental urine leakage that happens with coughing, sneezing and physical activity. Based on past research, current UK guidelines recommend that these women are offered at least three months of pelvic floor muscle exercises. The exercises are taught by a specialist physiotherapist or nurse. There is evidence that these exercises can strengthen the muscles and decrease leakage. It is not clear how "intensively" women have to exercise to get a good result that lasts thus improving the woman's quality of life and reducing the likelihood of surgery. Many women have stress incontinence; that is, accidental urine leakage that happens with coughing, sneezing and physical activity. Based on past research, current UK guidelines recommend that these women are offered at least three months of pelvic floor muscle exercises. The exercises are taught by a specialist physiotherapist or nurse. There is evidence that these exercises can strengthen the muscles and decrease leakage. It is not clear how "intensively" women have to exercise to get a good result that lasts thus improving the woman's quality of life and reducing the likelihood of surgery. This research aims to find out whether the use of biofeedback can help to improve the results of pelvic floor muscle exercises in the short- and longer-term. Using a probe inserted into the vagina, biofeedback equipment shows women what their pelvic floor muscles do. Because the muscles are "inside" they are "hidden", and biofeedback allows women to "see" the muscles working as they exercise. "Seeing" the muscles working on the biofeedback screen might help with teaching the exercises and encourage women to continue doing their exercises. A graph can be printed out to show changes in the muscle strength over time. The women who receive biofeedback may exercise more because they are more confident about doing the exercises and the changes that the exercises can achieve.
	6b	Explanation for choice of comparators **Explained in paper** (page 6, lines 6-9)
Objectives	7	Specific objectives or hypotheses **Explained in paper** (page 6, lines 11-14)
Trial design	8	Description of trial design including type of trial (eg, parallel group, crossover, factorial, single group), allocation ratio, and framework (eg, superiority, equivalence, non inferiority, exploratory) **Explained in paper** (page 6, lines 17-21)

Methods: Participants, interventions, and outcomes

4

For peer review only

Study setting	9	Description of study settings (eg, community clinic, academic hospital) and list of countries where data will be collected. Reference to where list of study sites can be obtained **Explained in paper** (page 6, lines 18-19)
Eligibility criteria	10	Inclusion and exclusion criteria for participants. If applicable, eligibility criteria for study centres and individuals who will perform the interventions (eg, surgeons, psychotherapists) **Explained in paper** (page 9, lines 8-23)
Interventions	11a	Interventions for each group with sufficient detail to allow replication, including how and when they will be administered **Explained in paper** (page 6, line 32 – page 7, line 36)
	11b	Criteria for discontinuing or modifying allocated interventions for a given trial participant (eg, drug dose change in response to harms, participant request, or improving/worsening disease) **Explained in paper** (page 7, lines 3-10)
	11c	Strategies to improve adherence to intervention protocols, and any procedures for monitoring adherence (eg, drug tablet return, laboratory tests) **Explained in paper** (page 7, lines 38-45 and page 7, lines 49- page 8, line 1)
	11d	Relevant concomitant care and interventions that are permitted or prohibited during the trial **Explained in paper** (page 7, lines 16-17)
Outcomes	12	Primary, secondary, and other outcomes, including the specific measurement variable (eg, systolic blood pressure), analysis metric (eg, change from baseline, final value, time to event), method of aggregation (eg, median, proportion), and time point for each outcome. Explanation of the clinical relevance of chosen efficacy and harm outcomes is strongly recommended **Explained in paper** (page 7, line 47 – page 8, line 33)
Participant timeline	13	Time schedule of enrolment, interventions (including any run-ins and washouts), assessments, and visits for participants. A schematic diagram is highly recommended (see Figure) **See SPIRIT figure**
Sample size	14	Estimated number of participants needed to achieve study objectives and how it was determined, including clinical and statistical assumptions supporting any sample size calculations **Explained in paper** (page 10, lines 32-37)
Recruitment	15	Strategies for achieving adequate participant enrolment to reach target sample size **Explained in paper** (page 8, line 45 – page 9, line 1)

Methods: Assignment of interventions (for controlled trials)

Allocation:

Sequence generation	16a	Method of generating the allocation sequence (eg, computer-generated random numbers), and list of any factors for stratification. To reduce predictability of a random sequence, details of any planned restriction (eg, blocking) should be provided in a separate document that is unavailable to those who enrol participants or assign interventions **Explained in paper**

(page 9, lines 25-30)

For peer review only

For peer review only

Allocation concealment mechanism	16b	Mechanism of implementing the allocation sequence (eg, central telephone; sequentially numbered, opaque, sealed envelopes), describing any steps to conceal the sequence until interventions are assigned **Explained in paper**
	(page 9, lines 26-30)	
Implementation	16c	Who will generate the allocation sequence, who will enrol participants, and who will assign participants to interventions **Explained in paper**
(page 9, lines 28-29)		
Blinding (masking)	17a	Who will be blinded after assignment to interventions (eg, trial participants, care providers, outcome assessors, data analysts), and how **Explained in paper**
	(page 9, lines 32-36)	
	17b	If blinded, circumstances under which unblinding is permissible, and procedure for revealing a participant's allocated intervention during the trial **Not applicable**
**Methods: Data collection, management, and analysis**		
Data collection methods	18a	Plans for assessment and collection of outcome, baseline, and other trial data, including any related processes to promote data quality (eg, duplicate measurements, training of assessors) and a description of study instruments (eg, questionnaires, laboratory tests) along with their reliability and validity, if known. Reference to where data collection forms can be found, if not in the protocol **Explained in paper**
	(page 7, line 47 - page 8, line 33)	
	18b	Plans to promote participant retention and complete follow-up, including list of any outcome data to be collected for participants who discontinue or deviate from intervention protocols **See trial protocol V6.0**
Data management	19	Plans for data entry, coding, security, and storage, including any related processes to promote data quality (eg, double data entry; range checks for data values). Reference to where details of data management procedures can be found, if not in the protocol **Data checking guidelines implemented by Trial Office.**
Statistical methods	20a	Statistical methods for analysing primary and secondary outcomes. Reference to where other details of the statistical analysis plan can be found, if not in the protocol **Explained in paper** (page 10, lines 4-18)
	20b	Methods for any additional analyses (eg, subgroup and adjusted analyses) **Explained in paper** (page 10, lines 18-20)
	20c	Definition of analysis population relating to protocol non-adherence (eg, as randomised analysis), and any statistical methods to handle missing data (eg, multiple imputation) **Explained in paper** (page 10, lines 15-18)
**Methods: Monitoring**		

For peer review only

6

For peer review only

Data monitoring	21a	Composition of data monitoring committee (DMC); summary of its role and reporting structure; statement of whether it is independent from the sponsor and competing interests; and reference to where further details about its charter can be found, if not in the protocol. Alternatively, an explanation of why a DMC is not needed **See trial protocol V6.0**
	21b	Description of any interim analyses and stopping guidelines, including who will have access to these interim results and make the final decision to terminate the trial **Explained in paper** (page 10, lines 5-7)
Harms	22	Plans for collecting, assessing, reporting, and managing solicited and spontaneously reported adverse events and other unintended effects of trial interventions or trial conduct **Explained in paper** (page 10, lines 39-44)
Auditing	23	Frequency and procedures for auditing trial conduct, if any, and whether the process will be independent from investigators and the sponsor **The TSC and DMEC will meet annually during the trial. An independent Chair and members will be appointed by the funder.**

Ethics and dissemination

Research ethics approval	24	Plans for seeking research ethics committee/institutional review board (REC/IRB) approval West of Scotland Research Ethics Committee 4, obtained on the 13th March 2013-13/WS/0048
Protocol amendments	25	Plans for communicating important protocol modifications (eg, changes to eligibility criteria, outcomes, analyses) to relevant parties (eg, investigators, REC/IRBs, trial participants, trial registries, journals, regulators) **Funders, sponsors and R&D will be notified routinely and appropriate approvals gained**
Consent or assent	26a	Who will obtain informed consent or assent from potential trial participants or authorised surrogates, and how (see Item 32) **Explained in paper** (page 9, lines 5-6)
	26b	Additional consent provisions for collection and use of participant data and biological specimens in ancillary studies, if applicable **Not applicable**
Confidentiality	27	How personal information about potential and enrolled participants will be collected, shared, and maintained in order to protect confidentiality before, during, and after the trial **Explained in paper** (page 9, line 38 – page 10, line 1)
Declaration of interests	28	Financial and other competing interests for principal investigators for the overall trial and each study site **None declared**

For peer review only - http://bmjopen.bmj.com/site/about/guidelines.xhtml

Access to data	29	Statement of who will have access to the final trial dataset, and disclosure of contractual agreements that limit such access for investigators **Explained in paper** (page 9, lines 40-41)
Ancillary and post-trial care	30	Provisions, if any, for ancillary and post-trial care, and for compensation to those who suffer harm from trial participation **N/A**
Dissemination policy	31a	Plans for investigators and sponsor to communicate trial results to participants, healthcare professionals, the public, and other relevant groups (eg, via publication, reporting in results databases, or other data sharing arrangements), including any publication restrictions **Explained in paper** (page 11, lines 9-12)
	31b	Authorship eligibility guidelines and any intended use of professional writers **OPAL Authorship Policy V2**
	31c	Plans, if any, for granting public access to the full protocol, participant-level dataset, and statistical code **To be discussed and agreed by the PMG and TSC**
**Appendices**		
Informed consent materials	32	Model consent form and other related documentation given to participants and authorised surrogates **Attached as an appendix**
Biological specimens	33	Plans for collection, laboratory evaluation, and storage of biological specimens for genetic or molecular analysis in the current trial and for future use in ancillary studies, if applicable **Not applicable**

For peer review only

*It is strongly recommended that this checklist be read in conjunction with the SPIRIT 2013 Explanation & Elaboration for important clarification on the items. Amendments to the protocol should be tracked and dated. The SPIRIT checklist is copyrighted by the SPIRIT Group under the Creative Commons "Attribution-NonCommercial-NoDerivs 3.0 Unported" license.

For peer review only
